# Comparative genomics highlights the unique biology of Methanomassiliicoccales, a Thermoplasmatales-related seventh order of methanogenic archaea that encodes pyrrolysine

**DOI:** 10.1186/1471-2164-15-679

**Published:** 2014-08-13

**Authors:** Guillaume Borrel, Nicolas Parisot, Hugh MB Harris, Eric Peyretaillade, Nadia Gaci, William Tottey, Olivier Bardot, Kasie Raymann, Simonetta Gribaldo, Pierre Peyret, Paul W O’Toole, Jean-François Brugère

**Affiliations:** EA-4678 CIDAM, Clermont Université, Université d’Auvergne, 28 Place Henri Dunant, BP 10448, 63000 Clermont-Ferrand, France; School of Microbiology and Alimentary Pharmabiotic Centre, University College Cork, Cork, Ireland; CNRS, UMR 6023, Université Blaise Pascal, 63000 Clermont-Ferrand, France; GReD, CNRS, UMR 6293, Inserm, UMR 1103, Clermont Université, Université d’Auvergne 28 Place Henri Dunant, BP 10448, 63000 Clermont-Ferrand, France; Département de Microbiologie, Unité de Biologie Moléculaire du Gène chez les Extrêmophiles, Paris Cedex 15, 75724 France; Cellule Pasteur UPMC, Université Pierre et Marie Curie, Paris Cedex 15, 75724 France

**Keywords:** Archaea, Methanomassiliicoccales, *Methanomethylophilus*, *Methanomassiliicoccus*, Origin of replication (ORI) binding (ORB) motif, Genome streamlining, CRISPR, Pyrrolysine Pyl, H_2_-dependent methylotrophic methanogenesis, Energy conservation

## Abstract

**Background:**

A seventh order of methanogens, the Methanomassiliicoccales, has been identified in diverse anaerobic environments including the gastrointestinal tracts (GIT) of humans and other animals and may contribute significantly to methane emission and global warming. Methanomassiliicoccales are phylogenetically distant from all other orders of methanogens and belong to a large evolutionary branch composed by lineages of non-methanogenic archaea such as Thermoplasmatales, the Deep Hydrothermal Vent Euryarchaeota-2 (DHVE-2, *Aciduliprofundum boonei*) and the Marine Group-II (MG-II). To better understand this new order and its relationship to other archaea, we manually curated and extensively compared the genome sequences of three Methanomassiliicoccales representatives derived from human GIT microbiota, “*Candidatus* Methanomethylophilus alvus", “*Candidatus* Methanomassiliicoccus intestinalis” and *Methanomassiliicoccus luminyensis*.

**Results:**

Comparative analyses revealed atypical features, such as the scattering of the ribosomal RNA genes in the genome and the absence of eukaryotic-like histone gene otherwise present in most of Euryarchaeota genomes. Previously identified in Thermoplasmatales genomes, these features are presently extended to several completely sequenced genomes of this large evolutionary branch, including MG-II and DHVE2. The three Methanomassiliicoccales genomes share a unique composition of genes involved in energy conservation suggesting an original combination of two main energy conservation processes previously described in other methanogens. They also display substantial differences with each other, such as their codon usage, the nature and origin of their CRISPRs systems and the genes possibly involved in particular environmental adaptations. The genome of *M. luminyensis* encodes several features to thrive in soil and sediment conditions suggesting its larger environmental distribution than GIT. Conversely, “*Ca.* M. alvus” and “*Ca.* M. intestinalis” do not present these features and could be more restricted and specialized on GIT. Prediction of the *amber* codon usage, either as a termination signal of translation or coding for pyrrolysine revealed contrasted patterns among the three genomes and suggests a different handling of the Pyl-encoding capacity.

**Conclusions:**

This study represents the first insights into the genomic organization and metabolic traits of the seventh order of methanogens. It suggests contrasted evolutionary history among the three analyzed Methanomassiliicoccales representatives and provides information on conserved characteristics among the overall methanogens and among Thermoplasmata.

**Electronic supplementary material:**

The online version of this article (doi:10.1186/1471-2164-15-679) contains supplementary material, which is available to authorized users.

## Background

Methanogenic archaea are distributed worldwide in anaerobic environments and account for a large proportion of methane emissions into the atmosphere, partly due to anthropogenic activity (*e.g.* rice fields and livestock). Over the last ten years, sequences of novel archaeal lineages distantly related to all orders of methanogens have recurrently been found in diverse anaerobic environments. One of these lineages, phylogenetically related to the Thermoplasmatales, was first reported in the rumen [1, 2] and was thereafter referred as Rumen Cluster-C in this environment [3]. The methanogenic nature of these archaea was subsequently strongly supported by the co-occurrence in human stool samples of 16S rRNA affiliated to this lineage and *mcrA* genes (a functional marker of methanogens) distantly related to any other methanogens [4, 5]. The final evidence that they represent a new order of methanogens was recently given with the isolation of *Methanomassiliicoccus luminyenis B10* from human feces [6] and the culture in consortia of several strains of this order: “*Candidatus* Methanomethylophilus alvus” [7] and “*Candidatus* Methanomassiliicoccus intestinalis” [8] from human feces samples, MpT1 and MpM2 [9] from termite gut and “*Candidatus* Methanogranum caenicola” [10] from waste treatment sludge. All the culture-based studies agreed on a common methanogenic pathway relying on the obligate dependence of the strains on an external H_2_ source to reduce methyl-compounds into methane. The restriction to this metabolism was previously only observed in two methanogens from digestive tract (*Methanosphaera stadtmanae* and *Methanomicrococcus blatticola*) and considered an exception [11]. The apparently large distribution of this obligate metabolism among this novel order of methanogens turns this exception into one of the important pathways among the overall methanogens. It also highlights the need for a more cautious utilisation of the term of “hydrogenotrophic methanogens” which is generally used to refer to methanogens growing on H_2_ + CO_2_, but also fits for an increasing number of described methanogens growing on H_2_ + methyl-compounds. Two names were proposed for this order, Methanoplasmatales [9] and Methanomassiliicoccales [10], the latter being now validated by the International Committee on Systematics of Prokaryotes [12]. For this reason, the name of Methanomassiliicoccales will be used in the current publication to refer to this novel order of methanogens.

The global contribution of Methanomassiliicoccales representatives to methane emission could be large, considering that it constitutes one of the three dominant archaeal lineages in the rumen [3] and in some ruminants it represents half or more of the methanogens [13–15]. Using *mcrA* and 16S rRNA sequences, several studies have also highlighted the broad environmental distribution of this order, not limited to digestive tracts of animals but also retrieved in rice paddy fields, natural wetlands, subseaflor and freshwater sediments for example [9, 10, 16, 17]. Methanomassiliicoccales were split into three large clusters, the “*Ca*. M. alvus” cluster, grouping sequences mostly retrieved from digestive tract of animals, the *M. luminyensis* cluster, mainly composed of sequences from soils and sediments and to a lesser extent from digestive tracts, and the Lake Pavin cluster formed by sequences retrieved from diverse environments but not digestive tracts [16].

The genome sequences of three different Methanomassiliicoccales members cultured from human stool samples, *M. luminyensis* B10 [18], “*Ca.* M. intestinalis Mx1-Issoire” [8] and “*Ca*. M. alvus Mx1201” [7], have recently been made available [19]. *M. luminyensis* shows 98% identity with “*Ca.* M. intestinalis” over the whole 16S rRNA gene and only 87% with “*Ca.* M. alvus”. According to the environmental origin of the sequences constituting the large cluster to which they belong, *M. luminyensis* and “*Ca.* M. intestinalis” might be more recently adapted to gut condition than “*Ca.* M. alvus”. Moreover the important difference in genome size and [G + C] % content between the two *Methanomassiliicoccus* spp. genomes suggests a rapid evolution of one of them in response to its adaptation from soil or sediment to digestive tract conditions [8]. Despite the important phylogenetic distance between “*Ca.* M. alvus” and the *Methanomassiliicoccus* spp., these genomes uncover common unique genomic characteristics. In particular, the analysis of “*Ca.* M. alvus” and *M. luminyensis* methanogenic pathways revealed they lack the 6 step C_1_-pathway forming methyl-CoM by the reduction of CO_2_ with H_2,_ otherwise present in all previously sequenced methanogens, fitting with their restriction to H_2_-dependent methylotrophic methanogenesis [16]. Moreover, these analyses helped define putative alternative substrates to methanol by identification of genes involved in the use of methylated-amines and dimethyl-sulfide. Methylated-amines utilization by Methanomassiliicoccales representatives has also been proposed in a metatranscriptomic study on rumen methanogens [17]. The use of tri-, di- and monomethylamine, with the obligate dependence on H_2_, has subsequently been validated *in vivo* with *M. luminyensis* [20]. This property could be significant for human health since gut-produced TMA could be implied in two different diseases [19–22]. The presence of pyrrolysine (Pyl, O), the 22^nd^ proteinogenic amino acid, is associated to this metabolism as it is incorporated in methyltransferases involved in utilization of methylated-amines through an *amber* codon suppression by a Pyl-tRNA [23, 24]. All the necessary genetic machinery is found in the three genomes of the Methanomassiliicoccales, including the genes for pyrrolysine synthesis (pylBCD), the *amber* suppressor tRNA^Pyl^ (pylT) and the dedicated amino-acyl tRNA synthetase (pylS). Their structure and unusual features, together with the evolutionary implications of this system have been recently described elsewhere [25].

These original metabolic and genetic characteristics, as well as the closer phylogenetic proximity of this order with Thermoplasmatales than other orders of methanogens prompted us to perform a more comprehensive analysis of these three genomes. We provide here their general characteristics, including comparisons to phylogenetic neighbor genomes, and derived potential metabolism and adaptation to environmental conditions from their gene composition. In the particular context of the missing genes of the CO_2_ reduction-pathway otherwise shared by all other methanogens, we reevaluate the global core of enzymes that are unique and specific to all methanogens and highlight the atypical composition of genes likely involved in energy conservation. The potential usage of the *amber* codon as a translational stop signal or encoding a Pyl in proteins was analyzed and suggests a differential handling of the Pyl-encoding capacity among the three Methanomassiliicoccales representatives.

## Results and discussion

### General genomic features

Genome size, [G + C] %, CDS and tRNA numbers were separately reported in the announcement of these genomes [7, 8, 18]. Data are gathered in the Table 1 with other newly defined general features.

**Table 1:** **Genome statistics**

Feature	“*Ca*. M. alvus”	“*Ca*. M. intestinalis”	*M. luminyensis*
Genome size^a^	1,666,795	1,931,651	2,637,810^d^
			(2,620,233)
DNA G + C content	55.6%	41.3%	60.5%
% DNA coding region	89.5%	88.4%	87.6%
Intergenic regions mean size (SD)^a^	102 (175)	119 (264)	121 (238)
Genes mean G + C content	56.3%	42.4%	61.0%
Putative replicons	1(+1)^b^	1(+1)^b^	1 (+1)^b^
Extrachromosomal elements	NA^c^	NA^c^	NA^c^
Total genes	1,705	1,882	2,713
RNA genes	52	50	52
rRNA genes (5S-16S-23S)	4 (2 - 1 - 1)	4 (2 - 1 - 1)	4 (2 - 1 - 1)
tRNA genes	48	46	48
Protein coding genes	1,653	1,832	2,661
Mean size of protein coding genes (SD)^a^	901 (667)	930 (890)	859 (676)
Median size of protein coding genes^a^	771	780	732
Gene products with function prediction	1,335	1,476	2,002
Gene products assigned to arCOGs	1,271	1,438	2,065
Gene products assigned Pfam domains	123	125	204
Gene products with signal peptides	247	336	512
Gene products with transmembrane helices	281	389	585
CRISPR repeats	1^e^	1	1

^a^Sizes are given in bp.

^b^Presence of two different *cdc6* genes per genome. See the text for more information.

^c^Not available.

^d^Data from [8]: in bracket stands the total bp (26 contigs) available from database [GenBank: CAJE01000001 to CAJE01000026], analyzed in this study.

^e^Presence of CRISPR repeats split into two neighboring loci (see Additional file 1: Table S3) surrounding a DNA sequence containing one gene encoding a putative transposase.

The tRNA gene complement present in the genomes is in part redundant and covers the usual 20 amino acids, with the exception of Lys in *M. luminyensis*, for which no tRNA was detected: this amino acid is likely encoded in the remnant ~17 kbp from this genome which are currently not available (Table 1). An archaeal complete set of amino-acyl tRNA synthetases is found in all three genomes, Asn- and Gln- tRNAs being obtained by an Asp-/Glu- tRNA (Asn/Gln) amidotransferase [26]. As previously described [25], an important feature is the presence of a tRNA^Pyl^ in all the three genomes. Several small non-coding RNAs (ncRNAs, complete list in Additional file 1: Table S1) were detected. Among them are found a Group II catalytic intron (only in “*Ca.* M. alvus”), the RNA component of the archaeal signal recognition particle (aSRP RNA) and the archaeal RNAse P.

**Figure 1:**
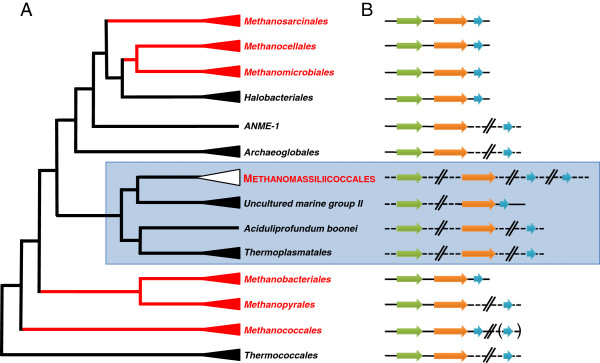
**Genomic features of ribosomal genes in Euryarchaeota. (A)** Phylogeny of Euryarchaeota highlighting the position of the Methanomassiliicoccales (according to [16]). The seven orders of methanogens are in red. **(B)** Genomic organization of ribosomal genes in Euryarchaeota: 5S, 16S and 23S rRNA genes are symbolized by blue, green and orange arrows, respectively. They are indicated irrespectively of the (+) or (-) DNA strand carrying them. A plain line defines an operon organization where tRNAs (when present) are not shown, nor the number of genes encoding rRNA with the exception of the Methanomassiliicoccales. The 5S rRNA gene in bracket refers to a second 5S rRNA copy isolated from the 16S-23S-5S rRNA gene operon in *Methanococcus maripaludis* C5.

Strikingly, 16S and 23S rRNA genes are not clustered and do not form a transcriptional unit as found in most bacterial and archaeal genomes. Among archaea, this unusual characteristic was first documented in Thermoplasmatales [27], but is also found in related lineages such as the uncultured Marine Group II (MG-II) and *Aciduliprofundum boonei* (Figure 1). This particular organization of the rRNA genes is consistent with the phylogenetic position of the seventh order of methanogens determined using a concatenation of ribosomal proteins [16] and constitutes a distinctive characteristic of Thermoplasmatales and related lineages. On a practical point of view, this also indicates that the Ribosomal Intergenic Transcribed Spacer Analysis, recently proposed as a tool to study the diversity of the methanogenic archaea in digesters [28], will likely fail to detect the Methanomassiliicoccales representatives.

As previously reported [8], the three genomes show significant size heterogeneity, with a variation of 58% (from around 1.7 Mbp to 2.6 Mbp, Table 1). Such heterogeneity is found even within the same genus with 36% size variation between the genomes of “*Ca.* M. intestinalis” and *M. luminyensis* (1.9 to 2.6 Mbp). The number of genes is highly variable and ranges from 1,705 (“*Ca.* M. alvus”) to 2,713 (*M. luminyensis*). The average CDS size and gene density is very close among the three genomes (around 900 bp and a protein coding gene every 984 to 1,054 bp). The main translation initiation codon is methionine (AUG) for which two copies of the corresponding tRNA are detected in “*Ca*. M. alvus” and three copies in *“Ca.* M. intestinalis” and *M. luminyensis*. In a lower extent, GUG and UUG are also found as translation start codons (Additional file 1: Table S2). Nucleotide composition [G + C] % ranges from 41.3% to 60.5% (Table 1) [7, 8, 18]. Codon usage patterns among CDS primarily reflect this [G + C] % variation, “*Ca.* M. intestinalis” primarily using AT-rich codons for a given amino acid (Additional file 1: Table S2). Two of the three stop codons follow this usage pattern, the *ochre* codon UAA accounts for 45% of the stop codons in the genome of “*Ca.* M. intestinalis” and respectively only 17% and 14% in the genomes of “Ca. M. alvus” and *M. luminyensis* and a same trend is observed for *opal* codon UGA (Additional file 1: Table S2). However, a different pattern is observed for the *amber* codon UAG and could be the result of a different selection process (see dedicated section on *amber* codon usage and putative Pyl-containing proteins). All the ribosomal RNA genes of the three genomes have a [G + C] % above 50%. In “*Ca.* M. intestinalis”, they thus have a largely higher [G + C] % than the genome average. When compared to *M. luminyensis*, general characteristics of the “*Ca.* M. intestinalis” genome suggest streamlining accompanied by a sharp [G + C] % reduction as previously observed in free-living *Prochlorococcus* [29]. This potential genomic evolution could be related to the recent colonization of digestive tract by “*Ca.* M. intestinalis” from soil or sediment environments.

### CRISPR elements

The CRISPR system confers to prokaryotes a highly adaptive and heritable resistance to foreign genetic elements such as plasmids and phages [30–33]. CRISPR loci are composed of genome-specific conserved Direct Repeats (DRs) separated by small sequences (spacers) which constitute a record of past infections. CRISPR-associated (Cas) proteins are responsible for integration of new spacers borrowed from invasive DNA and use the small antisense RNA transcript of these spacers to protect the cell from new invasions. CRISPR loci were previously notified in the three Methanomassiliicoccales genomes [7, 8, 18] and are characterized in the present study. The CRISPR DRs are concentrated in one genomic unit in *“Ca.* M. intestinalis” and *M. luminyensis* but are interrupted by a gene encoding a putative IS4-type transposase (AGI85628.1) in “*Ca.* M. alvus”. The DRs of the three genomes differ from each other in length (31 and 36 bp, Additional file 1: Table S3), sequence and associated 2D-structure (Additional file 2: Figure S1), and belong to three different superclasses. A CRISPR map analysis [34] attributed the *M. luminyensis* DRs to the superclass D, family 3 and the “*Ca.* M. intestinalis” DRs to the superclass A (no family) with a partial motif #27 which is exclusively shared with Methanococcales sequences (from *Methanothermus okinawensis*, *Methanocaldococcus jannaschii* and *Methanocaldococcus fervens*, Additional file 2: Figure S1). The “*Ca.* M. alvus” DRs (ATCTACACTAGTAGAAATTCTGAATGAGTTTTAGAC, superclass E) could not be classified in any sequence/structure family and likely represents a new family of CRISPR DR elements. The number of spacers within DRs ranges from 12 to 113 per locus (from 59 to 113 per genome). Each spacer has a particular size range, from 25 to 28 bp in “*Ca.* M. alvus” to 35 to 40 bp in *M. luminyensis* (Additional file 1: Table S3). A few other CRISPR-like elements are also found in as many as three copies and their functional role remains unknown (Additional file 1: Table S3).

According to the CRISPR system classification proposed by Makarova *et al*. [35] on the basis of organization and composition of the Cas protein-coding genes found in the neighborhood of the CRISPRs*, M. luminyensis* presents a CRISPR-Cas system subtype I-C (WP_019177384.1 to WP_019177390.1)*.* The CRISPR-Cas system of “*Ca.* M. intestinalis” is a hybrid of the subtypes I-A and I-B since its organization corresponds to subtype I-B, but contains the signature gene of the subtype I-A (Cas8a) (AGN26276 to AGY50180.1). The recently defined PreFran subtype (for Prevotella and Francisella) is present in “*Ca.* M. alvus” (AGI85629 to AGI85632). Notably, the Cas1 protein of “*Ca.* M. alvus” is predicted to contain a pyrrolysine (see section on *amber* codon usage and putative Pyl-containing proteins).

As suggested by the different superclass assignments of the repeats and the different types of CRISPR-Cas system, these CRISPRs likely result from non-vertical inheritance among the three species. The PreFran type, only found in 20 bacterial genomes so far is rather uncommon in comparison to the type I of the *Methanomassiliicoccus* spp. Bacteria that hold the PreFan type are generally found in tight association with animals and the genus *Prevotella* is one of the dominant in rumen [36] and human gut [37] suggesting that “*Ca.* M. alvus” may have acquired this system through other gut bacteria. Moreover, the spacers are specific to each of the three genomes suggesting they undergone different histories of infection. In “*Ca.* M. alvus”, one of the spacers is 93% similar (25 of 27 nt) to a ssDNA virus isolated from pig feces (JX305998.1).

With the exception of viruses from the families of *Myoviridae* and *Siphoviridae* (head-tail viruses) which also infect bacteria, archaeal viruses sequenced to date have almost no significant residue identity with each other and sequences in public databases [38, 39]. Accordingly, the lack of detection of prophage sequences by dedicated software does not imply the absence of prophages in these three genomes: some clusters of 10-30 adjacent genes with few significant matches in public databases might represent still unknown prophages. Furthermore, genes distantly related to phage ones are found in the three genomes and could belong to unknown prophages or represent residual traces of past infection. This is for example the case of two contiguous genes, present in the vicinity of the “*Ca.* M. intestinalis” CRISPR locus, which encode putative proteins (YP_008071639.1 & YP_008071640.1) with similarity to phage capsid synthesis proteins.

### Genome replication

Origins of replication were identified with a consensus Origin Recognition Box (ORB) motif recently identified from active replication origins of Thaumarchaeota (*Nitrosopumilus maritimus*)*,* Crenarchaeota and Euryarchaeota [40]. Several ORB motifs were found in the three genomes, most of them gathered by pairs (Table 2). A consensus sequence for a Methanomassiliicoccales ORB motif was deduced and shows little difference with the archaeal consensus recently proposed [40] (Table 2).

**Table 2:** **ORBs motifs found in the Methanomassiliicoccales genomes**

ORB	Sequence	Position	Spacing	Orientation	Comment
“*Ca*. M. alvus” ORB1	**GTTCCAGTGGAAATGG**-T**GGGGT**	78 - 99	39	inverted	downstream *orc1*/*cdc6.1*
“*Ca*. M. alvus” ORB2	**GTTCCA**C**TGGAAA**CA**G**-**AGGGGT**	138 - 159		inverted	downstream *orc1*/*cdc6.1*
“*Ca*. M. alvus” ORB3	T**TTCCA**C**TGGAAA**CA**G**-**AGGGGT**	1977 - 1998	47		upstream *orc1*/*cdc6.1*
“*Ca*. M. alvus” ORB4	**GTTCCA**C**TGGAAATGG**-T**GGGGT**	2045 - 2066			upstream *orc1*/*cdc6.1*
“*Ca*. M. intestinalis” ORB1	A**TT**A**CAGTGGAAATGA**-**AGGGGT**	15 - 36	256	inverted	downstream *orc1*/*cdc6.1*
“*Ca*. M. intestinalis” ORB2	T**TT**G**CAGTGGAAATGA**-**AGGGGT**	292 - 313			downstream *orc1*/*cdc6.1*
“*Ca*. M. intestinalis” ORB3^a^	**GTT**C**CAGTGGAAATGA**-**AGGGGT**	795626 - 795647			downstream *fstZ*
“*Ca*. M. intestinalis” ORB4^a^	TC**T**G**CA**C**TGGAAATGA-AGGGGT**	1576211 -1576232		inverted	downstream fused *nifH/nifE*
*M. luminyensis* ORB1	**GTT**C**CA**T**TGGAAAT**C**G**-GCA**GG**A	73488 - 73475^b^	113		downstream *orc1*/*cdc6.1*
*M. luminyensis* ORB2	**GTT**C**CAGTGGAAAT**A**A**-**AGGGGT**	73341 - 73362^b^		inverted	downstream *orc1*/*cdc6.1*
Methanomassiliicoccales consensus ORB	**GTTCCAGTGGAAATGG-AGGGGTA**				
Archaea consensus ORB	C**TTCCAGTGGAAA**C**G**AA**AGGGGT**				Pelve *et al*., [40]

Bases in bold indicate consensual bases of the ORB sequence in the Methanomassiliicoccales. The “*Ca*. M. alvus” ORBs, and the ORB2 of *M. luminyensis* and “*Ca*. M. intestinalis” might be extended by a “GGGGGT” sequence otherwise not conserved in the 4 other Methanomassiliioccales ORBs and the Archaea consensus ORB.

^a^Not found in close association to another ORB.

^b^Contig [GenBank: CAJE01000021.1].

**Figure 2:**
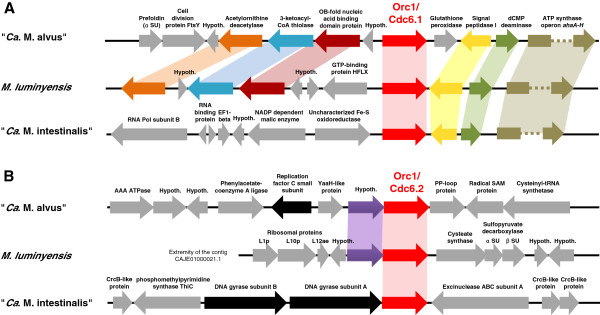
**Genomic regions surrounding the** ***orc1/cdc6.1*** **(A) and** ***orc1/cdc6.2*** **(B) genes in the three genomes of Methanomassiliicoccales.** Each homologous gene (*i.e.* showing more than 30% amino acid identity and an e-value < 10^-5^ when analyzed by blast against each other) from the 2 regions of the 3 genomes is colored differently and connected with shading. The black arrows represent genes involved in the replication process. The grey arrows represent other genes of various function with no homologue detected on the corresponding region of the 2 other genomes. “Hypoth.” refers to genes encoding hypothetical proteins.

Each of the three genomes possesses two copies of the *orc1/cdc6* (Origin Recognition complex/Cell division cycle 6) gene (Table 3). At least two ORB motifs are found in the vicinity of only one of the two *orc1*/*cdc6* genes. In the draft genome of *M. luminyensis*, these two genes are associated in the same contig (CAJE01000021), allowing comparison with the other two genomes. In every case, the *orc1*/*cdc6* genes are each located on a different strand (Additional file 2: Figure S2). They are close together within the *M. luminyensis* and “*Ca.* M. intestinalis” genomes (respectively around 70 and 90 kbp), and more distant in “*Ca.* M. alvus” (around 695 kbp). They are inversely oriented in the three genomes. Consistent with a recent study [41], phylogenetic analysis reveals that these genes correspond to two paralogs, *orc1/cdc6.1* and *orc1*/*cdc6.2* (Additional file 2: Figure S3). *orc1/cdc6.1* lies close to the predicted origin of replication, displays a conserved genomic context (Figure 2) is slow-evolving and groups phylogenetically with Thermoplasmatales/DHVE2/uncultured Marine Group II (Additional file 2: Figure S3), consistent with vertical inheritance. On the other hand, *orc1/cdc6.2* copies display much faster evolutionary rates, lies in a non-conserved genomic context (Figure 2), and show inconsistent phylogenetic placement close to Crenarchaeota (Additional file 2: Figure S3). This may be due to a tree reconstruction artifact or may represent a possible horizontal gene transfer from an unspecified crenarchaeon. Given its higher conservation, its conserved genomic context and its vicinity to ORB motifs, Orc1/Cdc6.1 is likely the main initiator protein and Orc1/Cdc6.2 may represent an inactive or accessory copy, possibly active in different environmental conditions.

**Table 3:** **DNA replication proteins compared to the corresponding components in Thermoplasmatales, MG-II and DHEV2**

	"*Ca*. M. alvus"	"*Ca*. M. intestinalis"	*M. luminyensis*	MG-II	DHEV2	Thermoplasmatales
ATP-dependent DNA ligase	AGI85913	AGN25909	WP_019176428	X	■	■
Orc1/Cdc6	AGI84758 (1)	AGN25419 (1)	WP_019178385 (1)	■■	■	■■
	AGI85775 (2)	AGN27158 (2)	WP_019178317 (2)			
DNA Pol D large subunit (DPL)	AGI85099	AGN26720	WP_019177373	■	■	■
DNA Pol D small subunit (DPS)	AGI84772	AGN27082	WP_019178373	■	■	■
FEN-1	AGI85207	AGN26626	WP_019176843	■	■	■
GINS 51	AGI84890	AGN27100	X	X	■	■
GINS 23	X	X	X	X	X	X
DNA Gyrase subunit B	[AGI86382]	[AGY50228]	[WP_019178436]	[■]	[■]	[■]
DNA Gyrase subunit A	[AGI86381]	[AGN27159]	[WP_019178437]	[■]	[■]	[■]
MCM	AGI86392	AGN26346	WP_019178416	■	■	■
		*AGN27203*				
PCNA	AGI84935	AGN27068	WP_019176118	■	■	■
DNA Pol B	AGI86264	AGN26701	WP_019177962	■	■	■
			*WP_019177491*			
Primase large subunit (PriL)	AGI84820	AGN27177	WP_019178297	■	■	■
Primase small subunit (PriS)	AGI86400	AGY50234	WP_019178400	■	■	■
RFC large subunit	AGI85559	AGN26596	WP_019176873	■	■	■
RFC small subunit	AGI85778	AGN26166	WP_019177244	■	■	■
RNaseH II	AGI86158	AGN25790	WP_019177553	■	■	■
TopoVI subunit A	AGI85998	AGN26743	WP_019177592	■	■	X
TopoVI subunit B	AGI85997	AGN26742	WP_019177591	■	■	X
Topo IB	X	X	X	X	X	X
SSB	X	X	X	X	■	■
RPA2	AGI84916	AGN25568	WP_019178149	■	■	■
		*AGY50184*	*WP_019177069*			
rpa2A (rp associated protein)	AGI84915	AGN25567	WP_019178150	■	■	■
NAD-dependent DNA ligase	[AGI85455]	X	X	[■]	X	X

Proteins in brackets indicate horizontal transfers from bacteria; Proteins in italics indicate fast evolving additional copies likely representing decaying paralogs, genes horizontally transferred among archaea, or homologs arising from integration of foreign elements. Absent proteins (or unavailable due to genome incompleteness) are indicated by an X. (1) and (2) in front of the Orc1/Cdc6 protein accession numbers indicate the Orc1/Cdc6.1 and Orc1/Cdc6.2, respectively.

The replication gene set is similar to that of the most closely related lineages (Table 3). However, some interesting features are present in the three genomes. For example, they do not harbor any homologs of the single-stranded binding protein SSB similarly to MG-II, whereas Thermoplasmatales and DHVE2 have both RPA and SSB. The absence of SSB may strengthen the sister relationship of the Methanomassiliicoccales and MG-II lineages as observed in a phylogenetic reconstruction based on ribosomal proteins [16]. The Methanomassiliicoccales, Marine Group II, and DHVE2 harbor both subunits of the archaeal topoisomerase TopoVI, strengthening a specific loss of this gene in Thermoplasmatales, which replaced it by a bacterial-type DNA gyrase [42]. Moreover, all three Methanomassiliicoccales representatives also harbor a bacterial-like DNA gyrase, known to have been acquired from bacteria in late emerging Euryarchaeota [41]. Some components are present as extra copy in the three genomes (in bold in Table 3), for example the Minichromosome Maintenance Protein (MCM) in the genome of “*Ca.* M. intestinalis”, which is highly divergent with respect to the other MCM coding genes and lies in a genome region with no synteny with the other closely related genomes. This is also the case for an extra PolB coding gene identified in the genome of *M. luminyensis*. Finally, genes coding for two additional OB-fold containing proteins (RPA-like) were identified in the genomes of “*Ca.* M. intestinalis” and *M. luminyensis*. All these extra copies are very divergent and likely represent decaying paralogs or homologs arising from integration of foreign elements. In addition, we found a bacterial type NAD-dependent DNA ligase homolog in the genome of “*Ca.* M. alvus” that appears to originate via a specific and recent horizontal gene transfer from a bacterium of the *Prevotella* genus, which is abundant in the human gut microbiota (Additional file 2: Figure S4A).

An important feature shared by the three Methanomassiliicoccales representatives, the Thermoplasmatales and other related lineages is the lack of Eukaryotic-like histone found in other Euryarchaeota [43], suggesting that the loss of this gene occurred early in the evolution of the whole lineage. Surprisingly, no gene coding for homologues of the bacterial-type HTa histones known to have replaced the native histone in Thermoplasmatales and DHVE2 are present in the Methanomassiliicoccales genomes and the MG-II genome. The DNA packaging function could be fulfilled in *M. luminyensis* by an Alba protein (WP_019176109.1) also presents in *Thermoplasmatales* [44] and MG-II, but absent in “*Ca.* M. alvus” and “*Ca.* M. intestinalis”. Few candidate proteins with a very weak similarity to bacterial histones and a Lys- and Arg-rich tail were identified in *M. luminyensis* (WP_019177894.1) and “*Ca.* M. intestinalis” (AGN26805.1) but not in “*Ca.* M. alvus”. While the proteins responsible for this crucial function remain elusive, a homologue of the histone acetyltransferase of the ELP3 family was identified in the three genomes (WP_019178580.1, AGN27049 and AGI86364). Only *M. luminyensis* possesses a histone deacetylase HdaI, related to Crenarchaeota and not found in other Thermoplasmatales (WP_019177579.1).

### Core genome

The best BLAST hits of the CDS from the three genomes were most frequently found in other archaeal members (70% to 82%), around 18% to 30% to Bacteria, and less than 0.3% to Eukaryota (Additional file 1: Table S4). It is likely that some of these reflect lateral gene transfer events, consistent with the presence of genomic islands with different [G + C] % composition from the genome average, as observed in “*Ca.* M. alvus” and, more pronounced, in “*Ca.* M. intestinalis” (Additional file 2: Figure S2).

**Figure 3:**
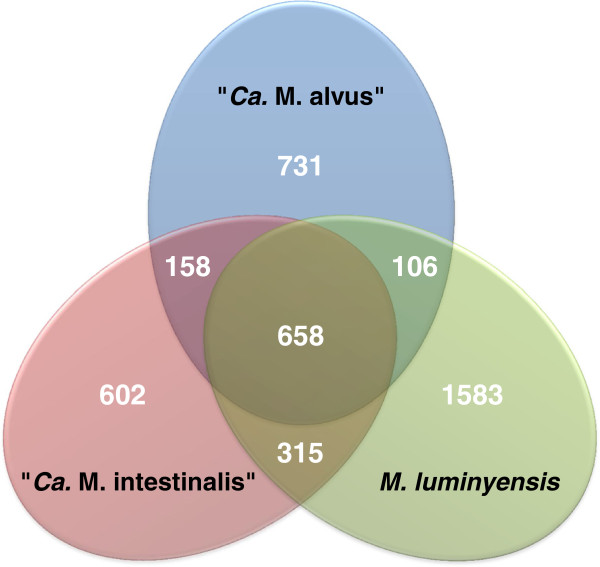
**Shared and unique CDS among the three genomes.** Venn diagram indicating the core genome at its center, deduced from a BLAST analysis of the CDS from the 3 genomes of the Methanomassiliicoccales. Unique and shared CDS among genome pairs are also given.

The core genome of the three species is composed of 658 CDS. While the number of CDS shared between genome pairs reflects partly their phylogenetic relatedness, an impressive proportion of CDS are specific to each one, in particular for *M. luminyensis* (Figure 3, Additional file 1: Table S5 for a complete list). Of the core genome, 173 genes are not found in the closest lineages (*Ferroplasma acidarmanus*, *Thermoplasma acidophilum*, *Thermoplasma volcanium*, uncultured Marine Group II and *Aciduliprofundum boonei* (Table 4, complete data in Additional file 1: Table S5). A part of these genes could correspond to specific traits of the Methanomassiliicoccales, at least for 20 of them which have no close homologue sequence in the databases (Additional file 1: Table S5). Another part of these genes reflects the metabolic pathway of the Methanomassiliicoccales representatives, methanogenesis, not shared with the Thermoplasmatales and any of the other related lineages for which genomic or physiological data are available. As discussed below, some of these genes are unique to methanogens. Among the predicted core proteins, 227 have no homologues in the two other methanogens commonly found in the same environment, the human gut (*Methanobrevibacter smithii* and *Methanosphaera stadtmanae*). Some of these differences rely on the particular methanogenic pathway of the Methanomassiliicoccales which can use methylated amines as substrate [20], which is not the case of *M. smithii* and *M. stadtmanae*. One hundred and two core proteins have no homologues in either the closely related lineages or the two gut methanogens (Table 4, complete data in Additional file 1: Table S6). Some show hits to other methanogens (Methanocellales, Methanomicrobiales and Methanosarcinales), and are specific for methanogenesis/energy conservation. Others likely reflect ancient lateral gene transfer events (LGTs) in the ancestor of the Methanomassiliicoccales. They include proteins involved in carbohydrate metabolism (glycosyl transferases, sugar transporters), nitrogen metabolism, and several proteins specific to the Methanosarcinaceae and involved in methanogenesis (see below).

**Table 4:** **Proteome of the three Methanomassiliicoccales representatives compared to their phylogenetic neighbors, human gut methanogens and NCBI nr proteins**

Core genome of Methanomassiliicoccales: 658 protein sequences	Specific ^a^	Shared ^b^
Phylogenetic neighbors	173	485
		*125 absent from human gut methanogens*
Human gut methanogens	227	431
		*63 absent from phylogenetic neighbors*
Phylogenetic neighbors and human gut methanogens	102	556 shared with at least one
		*Encompassing:*
		*125 absent from human gut methanogens*
		*71 absent from phylogenetic neighbors*
		360 shared with the two groups
NCBI non-redundant protein sequences database	20 (21)^c^	637

^a^Number of deduced proteins of the core genome of Methanomassiliicoccales that are not found in the corresponding organisms.

^b^Number of deduced proteins of the core genome of Methanomassiliicoccales that are also found in the corresponding organisms.

^c^The value of 21 encompasses CDS that are specific of the proteome of the Methanomassiliicoccales together with either the ones of the phylogenetic neighbors or of the human gut methanogens, without any other blast hits with the NCBI nr protein sequences database.

### General metabolism and adaptations to environment

Analysis of archaeal clusters of orthologous groups (ArCOG [45]) resulted in 1,271; 1,438 and 2,065 assigned functions for “*Ca.* M. alvus”, “*Ca.* M. intestinalis” and *M. luminyensis* respectively (representing between 77-79% of all CDS) (Additional file 1: Table S7). Components of cell wall/membrane and envelope biogenesis (class M) were less abundant when compared to the other gut methanogens *M. smithii* and *M. stadtmanae*. Indeed, comparatively to these Methanobacteriales, electron micrographies of *M. luminyensis* did not show a prominent cell-wall-like structure [6]. However, it seems that the synthesis of activated mannose is likely possible from fructose-6-P, therefore allowing the biosynthesis of N-glycans potentially associated to a cell-wall. A specific enrichment was observed for inorganic ion transport and metabolism (class P) and, as noted for other methanogens, for coenzyme transport and metabolism (class H): when analyzed in more details, many of the predicted transporters are ABC transporter permease proteins with homology to those identified in other methanogens (Additional file 1: Table S8). Noteworthy is the presence of quaternary ammonium compound efflux pumps as well as specialized systems involved in substrate acquisition for specialized methanogenesis-related functions (H_2_-dependent methylotrophic methanogenesis, see below): this includes putative transporters for dimethylamine (AGI85872.1/AGI85374.1/AGI85246.1 for “*Ca.* M. alvus”, AGN26255.1 for “*Ca.* M. intestinalis”, WP_019178528.1 for *M. luminyensis*) and trimethylamine (AGI85867.1, AGN26256.1, WP_019178522.1). The following part of the section focuses on several genomic features of the three Methanomassiliicoccales representatives that suggest metabolic adaptations to their environment. An overview of the inferred general metabolism is given in Additional file 2: Figure S5. As usually observed in methanogens, the three species harbors an incomplete reductive TCA cycle [46]. Further details on lipid, amino-acid and purine synthesis pathways, as well as molecular nitrogen fixation are also presented in Additional file 3.

Similarly to other methanogens and differently from the Thermoplasmatales representatives, the three Methanomassiliicoccales lack PurK for purine synthesis pathway. Two purE-like enzymes were identified (AGI84793.1, AGI85002.1, AGN25661.1, AGN26431.1, WP_019178351.1, WP_019177087.1) without clear assignment to class I or class II PurE (Additional file 3). Depending on the assignment of these PurE, the ATP-dependent activity of PurK might be substituted by a class I PurE in presence of high concentration of CO_2_ or a class II PurE, both avoiding the hydrolysis of ATP [47]. The former possibility could represent an adaptation to the high CO_2_ concentrations in anaerobic environments as proposed for other methanogens [47].

Two possible sources of ammonia are predicted to be common in the three Methanomassiliicoccales, a direct uptake from the environment by dedicated transporters (Additional file 1: Table S8) and an intracellular production, as a by-product of methanogenesis from monomethylamine. The presence of some of these transporters in close association to the genes involved in methanogenesis from monomethylamine suggests that they could alternatively be used to export ammonium when monomethylamine is used for methanogenesis. Ammonia could also be derived from urea in “*Ca*. M. intestinalis” which possesses a *ureA-G* operon encoding a urease (AGN27148.1 to AGN27154.1) and a urea transporter (AGN27055.1). Ammonia is likely assimilated by a glutamine synthetase GlnN, one in “*Ca*. M. alvus” and “*Ca*. M. intestinalis” (AGI86325.1; AGN25771.1) and two in *M. luminyensis* (WP_019177566.1; WP_019177539.1, this second one likely acquired through LGT from bacteria). *M. luminyensis* is predicted to be diazotroph with a putative flexibility upon the dependency on Molybdenum, while “*Ca.* M. alvus” and “*Ca.* M. intestinalis” probably lack the capacity to fix N_2_ (Additional file 3). N_2_ fixation capacity has been found among soil and sediment methanogens but not in common gut methanogens (Additional file 3) [48–50]. Accordingly, the potential capacity of *M. luminyensis* to fix N_2_ could reflect an adaptation to soil or sediment conditions and a facultative association to digestive tracts.

Each Methanomassiliicoccales genome encodes at least one catalase (*katE*), peroxiredoxin (*prx*), rubredoxin (*rub*) and rubrerythrin (*rbr*) to resist to oxygen exposure (Additional file 1: Table S9). *M. luminyensis* presents the highest antioxidant capacity, in particular with 8 copies of a peroxiredoxins (*prx*) gene, against 4 and 2 copies in “*Ca.* M. intestinalis” and “*Ca.* M. alvus” respectively. *M. luminyensis* is also the only one to harbor homologues of superoxide dismutase (*sodA*) and desulfoferrodoxin (*dfx*). A large diversity and redundancy of the antioxidant systems was previously reported for dominant rice field soil methanogens, Methanocellales, and described as a specific adaptation of these methanogens to oxic episodes regularly occurring in these environments [48, 49]. In line with its probable diazotrophic capacity, the larger number and diversity of genes encoding antioxidant enzymes in *M. luminyensis* argue for a greater adaptation to soil environments than “*Ca.* M. alvus” and “*Ca.* M. intestinalis”. A glycine-betaine ABC transporter (WP_019176328.1, WP_019176329.1, WP_019176330.1) was also found in *M. luminyensis*. This kind of transporter helps to cope with external variations in salt concentration by accumulating glycine-betaine as an osmoprotectant and was previously identified in Methanosarcinales [51, 52]. No similar transporter of glycine-betaine was identified in “*Ca.* M. alvus” or “*Ca.* M. intestinalis”.

Interestingly, among the three Methanomassiliicoccales representatives, “*Ca.* M. alvus” is the only one to encode a choloylglycine hydrolase (YP_007713843.1), which confers resistance to bile salts encountered in the gastro-intestinal tracts (GIT). This gene is also present in the genome of the two other dominant human gut methanogens, *M. smithii* and M*. stadtmanae* [53, 54], and could have been transferred from other gut bacteria (Additional file 2: Figure S4B). Another adaptation to GIT could be inferred through the presence of a conserved amino acid domain corresponding to COG0790 (TPR repeat, SEL1 subfamily) in at least one protein of each Methanomassiliicoccales representative. This conserved domain has been previously identified in proteins involved in interactions between bacteria and eukaryotes and was never reported in archaea [55] suggesting an adaptation to digestive tracts unique to Methanomassiliicoccales among archaea. In that case, the occurrence of the genes encoding proteins with this domain in the Methanomassiliicoccales genomes, 28 in “*Ca.* M. alvus”, 6 in “*Ca.* M. intestinalis” and one in *M. luminyensis* would support a higher adaptation of “*Ca.* M. alvus” to digestive tracts.

### Methanogenesis and core enzymes specific to methanogens

**Figure 4:**
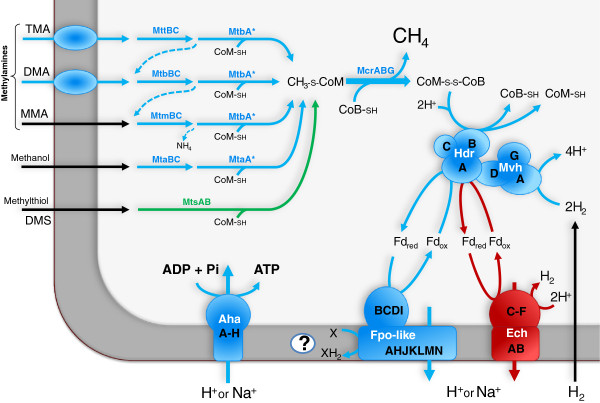
**Proposed pathways for methanogenesis and energy conservation in the Methanomassiliicoccales representatives.** The protein names are in bold. The predicted pathways and enzymes present in the three Methanomassiliicoccales species are in blue, those absent from "*Ca.* M. intestinalis" are in green and those absent from "*Ca.* M. alvus" are in red. MtaA and MtbA are marked with an asterisk to signify that the homologs present in the Methanomassiliicoccales are not yet assigned to one or the other enzyme category. "X" refers to the uncharacterized lipid soluble electron transporter. The question mark points out that the enzymes involved in the reoxidation of the lipid soluble electron transporter remain to be uncovered. See Table 6 and Additional file 1: Table S10 for a description of the set of genes involved.

It was previously reported that *M. luminyensis* and “*Ca.* M. alvus” lack the genes that encode the 6 step C_1_-pathway leading to methyl-CoM by the reduction of CO_2_ with H_2_ [16]. Our current analysis revealed a similar lack of these genes in “*Ca.* M. intestinalis” (Figure 4). It also reveals that “*Ca.* M. intestinalis” does not harbor the genes *mtsAB* (Figure 4) which code for enzymes likely involved in methanogenesis from dimethylsulfide [56]. The composition of the methyltransferases involved in the H_2_-dependent methylotrophic methanogenesis from the three genomes was partially determined before [7, 8, 16, 17] and is compiled in the Additional file 1: Table S10, with their relative genomic position displayed in the Additional file 2: Figure S6.

A pool of genes conserved among all methanogens and not found in any other archaea was recently determined by Kaster *et al.* [57]. These genes encode the subunits of two enzymatic complexes unique and shared by all methanogens, the methyl-H4MPT: coenzyme M methyltransferase (Mtr) and the methyl coenzyme reductase (two complexes of isoenzymes Mcr and Mrt), as well as proteins of unknown function. Being unique to methanogens, these uncharacterized proteins likely have an important role for methanogenesis and could be directly associated to the functioning of Mcr and Mtr [57]. The lack of Mtr and the other genes of the CO_2_-reductive pathway in the three Methanomassiliicoccales described here, prompted us to reevaluate the overall methanogenesis markers. In addition to the five genes coding for subunits of the Mtr enzymatic complex, two former methanogenesis markers (annotated as methanogenesis markers 10 and 14 in the databases and belonging to arCOG00950 and arCOG04866, respectively) are absent from the three Methanomassiliicoccales genomes (Table 5). One of these genes (belonging to arCOG04866) is present in the vicinity of the operon coding for Mtr in *Methanosaeta thermophila*, Methanobacteriales, Methanopyrales and Methanocellales genomes. Its genomic position in methanogens encoding Mtr and its absence in Methanomassiliicoccales suggests its involvement in the functioning of Mtr. Fifteen genes present in the three Methanomassiliicoccales genomes have homologues (and/or paralogs in the case of *atwA* and the *mcr/mrt* operons) conserved in all other methanogens and not in other archaea and could still be considered as methanogenesis markers (Table 5). Interestingly, 13 of these genes, including the *mcr* operon, are clustered on a small genomic portion (~16 Kb) of *M. luminyensis* and “*Ca.* M. alvus”. At the exception of *mcrABG* and *atwA* [58], they encode for proteins of unknown function. One of these proteins (WP_019176775.1, AGN26870, AGI85145), not previously reported as a methanogenesis marker, might be associated to the functioning of Mcr as it is encoded by a gene located directly upstream *mcrA* in the three Methanomassiliicoccales genomes. The nifD-like (NflD) gene previously proposed to be involved in the biosynthesis of the coenzyme F_430_, the prosthetic group of Mcr/Mrt, is also present in the three genomes [59]. It forms a cluster with a UDP-N-acetylmuramyl pentapeptide synthase like gene (Table 5) and a *nifH-like* gene also suggested to be involved in coenzyme F_430_ biosynthesis. Several uncharacterized proteins are shared by almost all methanogens, while present in very few other archaea, suggesting a tight relationship with methanogenesis (Table 5). This is for example the case of a putative methyltransferase MtxX [60] only missing in *Methanosaeta concilii* GP6 (but still present as a pseudogene, MCON_2260) among methanogens and only present in *Ferroglobus placidus* DSM-10642 among non-methanogens.

**Table 5:** **Core proteins of methanogenesis**

Annotation	"*Ca.*M. alvus"	"*Ca.*M. intestinalis"	*M. luminyensis*	Distribution	arCOG
Nitrogenase molybdenum-iron like protein (NifD-like/NflD)	*AGI86050*	*AGN27015*	*WP_019176684.1*	1	arCOG04888
UDP-N-acetylmuramyl pentapeptide synthase like protein (MurF-like)	*AGI86051*	*AGN27016*	*WP_019176685.1*	1	arCOG02822
Methyl-coenzyme M reductase operon associated like protein (McrC-like)	**AGI85157**	***AGN26013***	**WP_019176790.1**	1	arCOG03226
Conserved hypothetical protein	**AGI85156**	***AGN26012***	**WP_019176789.1**	1	arCOG04904
CoA-substrate-specific enzyme activase	**AGI85155**	***AGN26011***	**WP_019176788.1**	1	arCOG02679
Conserved hypothetical protein	**AGI85154**	***AGN26010***	**WP_019176787.1**	1	arCOG04903
Conserved hypothetical protein	**AGI85153**	***AGN26009***	**WP_019176786.1**	1	arCOG04901
Peptidyl-prolyl cis-trans isomerase related protein	**AGI85152**	***AGN26008***	**WP_019176785.1**	1	arCOG04900
Methyl coenzyme M reductase operon associated protein (McrC)	**AGI85151**	***AGN26006***	**WP_019176783.1**	1	arCOG03225
Methyl-coenzyme M reductase, component A2 (AtwA)	**AGI85150**	***AGN26005***	**WP_019176782.1**	1*^†^	arCOG00185
Methyl coenzyme M reductase, beta subunit (McrB/MrtB)	**AGI85141**	**AGN26874**	**WP_019176771.1**	1*	arCOG04860
Methyl coenzyme M reductase, protein D (McrD/MrtD)	**AGI85142**	**AGN26873**	**WP_019176772.1**	1*	arCOG04859
Methyl coenzyme M reductase, gamma subunit (McrG/MrtG)	**AGI85143**	**AGN26872**	**WP_019176773.1**	1*	arCOG04858
Methyl coenzyme M reductase, alpha subunit (McrA/MrtA)	**AGI85144**	**AGN26871**	**WP_019176774.1**	1*	arCOG04857
SH3 fold protein	**AGI85145**	**AGN26870**	**WP_019176775.1**	1	arCOG04846
Conserved hypothetical protein	**AGI85146**	**AGN26876**	**WP_019176769.1**	2*^†^	arCOG02882
AIR synthase-like protein	AGI85549	AGN26462	WP_019176932.1	2	arCOG00640
Predicted DNA-binding protein containing a Zn-ribbon domain	AGI84948	AGN25597	WP_019176187.1	2*	arCOG01116
Methyltransferase related protein (MtxX)	AGI85117	AGN26654	WP_019177314.1	3	arCOG00854
Conserved hypothetical protein	AGI84870	AGN25885	WP_019178690.1	3*	arCOG04893
Fe-S oxidoreductase, related to NifB/MoaA family	-	-	-	4*	arCOG00950
Conserved hypothetical protein	-	-	-	4	arCOG04866
N5-methyltetrahydromethanopterin: coenzyme M methyltransferase, subunit A (MtrA)	-	-	-	4*	arCOG03221
N5-methyltetrahydromethanopterin: coenzyme M methyltransferase, subunit B (MtrB)	-	-	-	4	arCOG04867
N5-methyltetrahydromethanopterin: coenzyme M methyltransferase, subunit C (MtrC)	-	-	-	4	arCOG04868
N5-methyltetrahydromethanopterin: coenzyme M methyltransferase, subunit D (MtrD)	-	-	-	4	arCOG04869
N5-methyltetrahydromethanopterin: coenzyme M methyltransferase, subunit E (MtrE)	-	-	-	4	arCOG04870
Soluble P-type ATPase	-	-	-	5	arCOG01579
Uncharacterized conserved protein	-	-	-	5*	arCOG04844
Conserved hypothetical protein (putative kinase)	-	-	-	6	arCOG04885

Protein accession numbers with the same font (bold, italics or bold-italics) are encoded by genes situated close to each other in their respective genomes.

*Paralogues.

^†^Related to a bacterial cluster with same conserved domain.

1, Methanogenesis marker, present in and unique to all sequenced methanogens and not in other archaea.

2, Present in all sequenced methanogens and less than 5% of other sequenced archaea.

3, Present in more than 90% of sequenced methanogens including Methanomassiliicoccales and less than 5% of other sequenced archaea.

4, Absent from the Methanomassiliicoccales but present and unique to all other methanogens.

5, Absent from the Methanomassiliicoccales but present in more than 90% other methanogens and not in other archaea.

6, Absent from the Methanomassiliicoccales but present in more than 90% of sequenced methanogens and less than 5% of other sequenced archaea.

Other genes present in the three genomes are more widely distributed than in methanogens but play a crucial role in methanogenesis. This is the case of genes required for the biosynthesis of the coenzyme M and coenzyme B involved in the last step of methanogenesis. Inferred CoM biosynthesis uses sulfopyruvate, which originates from 3-phosphoserine converted to cysteate by a cysteate synthase and then to sulfopyruvate (ComDE), as observed in Methanosarcinales, Methanomicrobiales [61] and Methanocellales (Additional file 1: Table S11). An alternative pathway takes place in other methanogens, where CoM originates from phosphoenolpyruvate and sulfite to produce sulfolactate, which is then oxidized [62–64]. These steps require the activity of enzymes encoded by the *comABC* genes which are absent in the three genomes, similar to what is observed in Methanosarcinales and Methanomicrobiales (Additional file 1: Table S11).

### Energy conservation

Methanogenesis is coupled to energy conservation through the establishment of a proton and/or sodium ion electrochemical gradient across the cytoplasmic membrane that drives an archaeal-type A_1_A_0_ ATP synthase complex to form ATP [65]. The genes coding for this complex are found in close association with the putative origin of replication in the three genomes (Figure 2, Table 6). The exergonic reduction of the heterodisulfide CoM-S-S-CoB formed by the Mcr complex is a crucial step for energy conservation conserved in all methanogens. The three genomes harbor at least one copy of *hdrA, hdrB* and *hdrC* homologues encoding a soluble heterodisulfide reductase (Table 6), HdrB representing the catalytic activity for CoM-S-S-CoB reduction. The current HdrA differs from its homologues present in other methanogens by its longer size and the presence of two predicted FAD-binding sites instead of one, and three 4Fe-4S centers instead of four. The three genomes also contain homologues of *hdrD,* encoding the catalytic site of a second class of heterodisulfide reductase (HdrDE), but no homologues of *hdrE* encoding the membrane bound cytochrome subunit of this complex. Similarly to the Methanococcales, Methanobacteriales and Methanopyrales, the *hdrB* and *hdrC* genes are adjacent whereas the *hdrA* gene is located apart and in close association with *mvhDGA* encoding the cytoplasmic F_420_-non-reducing hydrogenase, absent from members of the Methanosarcinales and some Methanomicrobiales [66]. MvhA contains the Ni-Fe domain for activation of H_2_. MvhADG and HdrABC were shown to form a complex that couples the reduction of CoM-S-S-CoB and a ferredoxin with H_2_ through a flavin-based electron bifurcation in *Methanothermobacter marburgensis* [67]. Presence of MvhADG and HdrABC in the three Methanomassiliicoccales representatives suggests a similar process (Figure 4). Energy conservation may likely result from the subsequent reoxidation of ferredoxin coupled to translocation of H^+^ (or possibly Na^+^) across the membrane by a membrane associated enzymatic complex (Figure 4), as proposed by Thauer *et al.* [68] for *M. stadtmanae*. However the Ehb complex likely responsible for the translocation Na^+^ in *M. stadtmanae* is not present in the three Methanomassiliicoccales representatives.

**Table 6:** **Genes involved in energy conservation in "** ***Ca*** **. M. alvus", "** ***Ca*** **. M. intestinalis" and** ***M. luminyensis*** **and accession numbers of the proteins they encode**

	"*Ca*. M. alvus"	"*Ca*. M. intestinalis"	*M. luminyensis*	Transmembrane helices
ATP synthase				
*ahaH*	AGI84762.1	AGN25422.1	WP_019178382.1	no
*ahaI*	AGI84763.1	AGN25423.1	WP_019178381.1	yes
*ahaK*	AGI84764.1	AGN25424.1	WP_019178380.1	yes
*ahaE*	AGI84765.1	AGN25425.1	WP_019178379.1	no
*ahaC*	AGI84766.1	AGN25426.1	WP_019178378.1	no
*ahaF*	AGI84767.1	AGN25427.1	WP_019178377.1	no
*ahaA*	AGI84768.1	AGN25428.1	WP_019178376.1	no
*ahaB*	AGI84769.1	AGN25429.1	WP_019178375.1	no
*ahaD*	AGI84770.1	AGN25430.1	WP_019178374.1	no
Membrane-bound proton-translocating pyrophosphatase				
*hppA*	/	AGN26077.1	WP_019176822.1	yes
Heterodisulfide reductase				
*hdrA*	AGI85054.1	AGN25863.1	WP_019177460.1	no
*hdrB1*	AGI86093.1	AGN25718.1	WP_019177711.1	no
*hdrB2*	AGI85474.1	AGN25916.1	WP_019176125.1	no
*hdrC1*	AGI86094.1	AGN25719.1	WP_019177712.1	no
*hdrC2*	/	/	WP_019176126.1	no
*hdrD1*	AGI86375.1	AGN25510.1	WP_019178460.1	no
*hdrD2*	AGI86212.1	AGN25649.1	WP_019177852.1	no
*hdrD3*	/	/	WP_019177557.1	no
*hdrE*	/	/	/	/
Methyl-viologen-reducing hydrogenase				
*mvhD1*	AGI85055.1	AGN25864.1	WP_019177459.1	no
*mvhD2*	/	AGN25453.1	WP_019176201.1	no
*mvhD3*	/	/	WP_019176130.1	no
*mvhG*	AGI85056.1	AGN25865.1	WP_019177458.1	no
*mvhA*	AGI85057.1	AGN25866.1	WP_019177457.1	no
F_420_H_2_ dehydrogenase-like/11-subunit respiratory complex 1				
*fpoA*	AHA34030.1	AGN25601.1	WP_019176183.1	yes
*fpoB*	AGI84952.1	AGN25602.1	WP_019176182.1	no
*fpoC*	AGI84953.1	AGN25603.1	WP_019176181.1	no
*fpoD*	AGI84954.1	AGN25604.1	WP_019176180.1	no
*fpoF*	/	/	/	
*fpoH*	AGI84955.1	AGN25605.1	WP_019176179.1	yes
*fpoI*	AGI84956.1	AGN25606.1	WP_019176178.1	no
*fpoJ*_*N*_	AGI84957.1	AGN25607.1	WP_019176177.1	yes
*fpoJ*_*C*_	AGI84958.1	AGN25608.1	WP_019176176.1	yes
*fpoK*	AGI84959.1	AGN25609.1	WP_019176175.1	yes
*fpoL*	AGI84960.1	AGN25610.1	WP_019176174.1	yes
*fpoM*	AGI84961.1	AGN25611.1	WP_019176173.1	yes
*fpoN*	AGI84962.1	AGN25612.1	WP_019176172.1	yes
*fpoO*	/	/	/	
Energy-converting hydrogenase				
*echA1*	/	AGN25511.1	WP_019178471.1	yes
*echA2*	/	AGN26997.1	WP_019176386.1	yes
*echB1*	/	AGN25512.1	WP_019178472.1	yes
*echB2*	/	AGN26998.1	WP_019176385.1	yes
*echC1*	/	AGN25513.1	WP_019178473.1	no
*echC2*	/	AGN26999.1	WP_019176384.1	no
*echD1*	/	AGN25514.1	WP_019178474.1	no
*echD2*	/	AGN27000.1	WP_019176383.1	no
*echE1*	/	AGN25515.1	WP_019178475.1	no
*echE2*	/	AGN27001.1	WP_019176382.1	no
*echF1*	/	AGN25516.1	WP_019178476.1	no
*echF2*	/	AGN27002.1	WP_019176381.1	no
Liposoluble electron transporter synthesis				
*ispA* ^a^	AGI84964.1	AGN25614.1	WP_019176170.1	/
*ubiA* ^b^	AGI85875.1	AGN26416.1	WP_019178349.1	/
		AGN26109.1		
*ubiE* ^c^	AGI85874.1	AGN26417.1	WP_019178072.1	/
		AGN25541.1	WP_019178198.1	
			WP_019176998.1	

^a^encoding a geranylgeranyl pyrophosphate synthase (GGPPS).

^b^encoding a1,4-dihydroxy-2-naphthoate octaprenyltransferase (DHNOPT).

^c^encoding a 2-heptaprenyl-1,4-naphthoquinone methyltransferase (HPNQMT).

The only identified complex shared by the three genomes which could fulfil this role corresponds to the 11-subunits respiratory complex I found in a large number of archaea and bacteria [69]. This complex is homologous to the Fpo complex (F_420_H_2_ dehydrogenase) of Methanosarcinales [70]. Characterized respiratory complex I and Fpo catalyze the exergonic transfer of electrons from a cytoplasmic electron transporter to a membrane soluble electron transporter coupled to the translocation of ions across the membrane [69, 70]. A similar process in Methanomassiliicoccales would thus imply a membrane associated electron transport chain which was so far only observed in *Methanosarcinales* among methanogens. The currently predicted enzymatic complex is truncated as compared to the Fpo of *Methanosarcina* spp. with the lack of homologues of the FpoO and FpoF subunits, forming an FpoABCDHIJKLMN like complex (Figure 4, Table 6). The lack of the FpoF subunit is similar to the Fpo complex of *Methanosaeta* representatives which were proposed to use ferredoxin instead of F_420_H_2_ as electron donor [71] (Table 6). The three genomes also harbor genes required for biosynthesis of a liposoluble electron transporter (Additional file 3, Table 6), whose role may be to accept electrons from the Fpo complex [72]. This membrane-soluble electron carrier, whose biochemical nature has to be determined experimentally, would drive electron transfer in the membrane, linking the Fpo complex to another membrane bound protein/complex, possibly a second coupling site reducing the heterodisulfide. The energy-converting hydrogenase EchA-F is another membrane enzymatic complex which could also translocate ions by the re-oxidation of the ferredoxin [73] but it only occurs in *M. luminyensis* and “*Ca.* M. intestinalis” (Figure 4). Nevertheless EchA-F could also operate in reverse and exploit the chemosmotic gradient for anabolic reactions [74]. Finally, a gene encoding a membrane-bound pyrophosphatase is found in the genomes of *M. luminyensis* and “*Ca.* M. intestinalis” (Table 6) but not in “*Ca.* M. alvus”. This protein is predicted to allow the translocation of protons across the cytoplasmic membrane by hydrolysis of PPi to phosphate [75, 76].

The three genomes share an original combination of genes likely involved in energy conservation, suggesting a different process than what is observed in other methanogens. The predicted flavin-based electron bifurcation in MvhADG/HdrABC complex is a feature shared by most methanogens with the exception of Methanosarcinales and some Methanomicrobiales representatives, while the putative membrane associated electron transport chain related to the activity of the Fpo-like complex was so far a unique feature of Methanosarcinales among methanogens. However, no membrane-bound cytochrome protein like those of the Methanosarcinales was detected to be encoded by the three genomes and the complete process remains to be uncovered.

### *Amber*codon usage and putative Pyl-containing proteins

**Figure 5:**
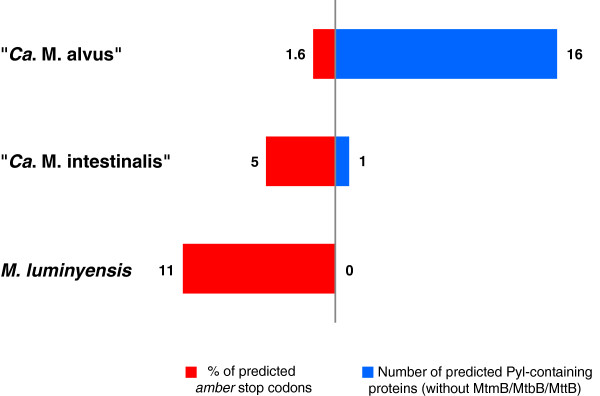
**Comparison of the number of putative Pyl-containing proteins (other than MtmB/MtbB/MttB methyltransferases) and of the percentage of** ***amber*** **codons used as translational stop signals deduced from the three Methanomassiliicoccales genomes.**

Previous studies have shown that the genes coding for methyl:corrinoid methyltransferases B dedicated to methylamines utilization (*mtmB*, *mtbB* and *mttB* for mono-, di- and tri-methylamines, respectively) present in *M. luminyensis*, “*Ca.* M. intestinalis” and “*Ca.* M. alvus” contain an in-frame *amber* Pyl-encoding codon [7, 8, 25], similarly to what is observed in Methanosarcinaceae and in a few bacteria [77, 78], where it encodes the 22^nd^ proteogenic amino acid pyrrolysine (Pyl, O). All the necessary genetic machinery is found in the three Methanomassiliicoccales genomes, including the genes for pyrrolysine synthesis (pylBCD), the *amber* suppressor tRNA^Pyl^ (pylT) and the dedicated amino-acyl tRNA synthetase (pylS) [25]. The presence of decoding *amber* machinery questions the occurrence of Pyl in other proteins than the methyltransferases involved in methylotrophic methanogenesis. This possibility was addressed in the present study by searching all the TAG-interrupted CDS which share the same BLASTP hit with the virtual in-frame translation of the 3’ flanking region. These CDS were fused *in silico* as a unique CDS, stopping at the next stop codon and predicted as potentially incorporating Pyl during the translation process. As a positive control, this strategy identified the above-mentioned methylamines:corrinoid methyltransferases in the three genomes. No putative other Pyl-containing proteins were identified in *M. luminyensis.* One additional *amber*-containing CDS was determined in “*Ca.* M. intestinalis”, a putative Fe-S binding protein (AGY50215), which is absent in “*Ca.* M. alvus” and present in *M. luminyensis* but not predicted to incorporate Pyl. “*Ca.* M. alvus” contains the highest number of predicted Pyl-containing proteins, 16 in addition to the methylamines: corrinoid methyltransferases (Table 7, Figure 5). Half of them have homologues in the two other genomes but without in-frame *amber* codons (in bold, Table 7). Among these 16 proteins, several have a hypothetical function and some are highly conserved in methanogens and/or archaea. This is the case of a digeranylgeranylglyceryl phosphate synthase required in the synthesis of archaeal phospholipids and of the putative methyltransferase MtxX (Tables 5 and 7). The CRISPR associated *cas1* gene, although present in the three genomes, is only detected as a Pyl-containing enzyme in “*Ca.* M. alvus”. The activity and the effective incorporation of Pyl in such a large range of enzymes of the same organisms remain to be determined experimentally. However, this could reasonably be assumed considering the existence of few functional Pyl-containing proteins (different of methylamines:corrinoid methyltransferases) reported from both Pyl-decoding archaea and bacteria [77, 79, 80].

**Table 7:** **Putative Pyl-containing proteins in "** ***Ca*** **. M. alvus"**

Accession number	Annotation	Size ^a^	Comments
**AGI84833.1**	hypothetical protein	253	DPM synthase like/GT2 superfamily
**AGI85009.1**	hypothetical protein	270	digeranylgeranylglyceryl phosphate synthase
**AGI85117.2**	phosphotransacetylase-like protein	242	putative methyltransferase MtxX
**AGI85168.2**	filamentation induced by cAMP protein Fic	425	
AGI85186.1	hypothetical protein	149	Rv0623-like transcription factor
AGI85280.1	hypothetical protein	917	glycosyltransferase family 29
AGI85290.1	hypothetical protein	148	
AGI85300.1	hypothetical protein	444	ATPase domain
AGI85437.1	hypothetical protein	536	prophage Lp3 protein 8 (helicase) of *Lactobacillus* spp.
AGI85443.1	hypothetical protein	717	
**AGI85449.1**	hypothetical protein	262	putative methyltransferase
AGI85596.1	hypothetical protein	162	putative acetyltransferase
AGI85630.1	hypothetical protein	322	CRISPR- associated endonuclease cas1
*AGI85862.1*	*MMA:corrinoid methyltransferase*	*459*	
*AGI85863.1*	*MMA:corrinoid methyltransferase*	*461*	
*AGI85869.1*	*TMA:corrinoid methyltransferase*	*504*	
*AGI85870.1*	*DMA:corrinoid methyltransferase*	*469*	
AGI86303.1	hypothetical protein	389	Sel-1 domain containing protein
AGI86346.1	transporter family protein	289	bacterial/archaeal transporter family protein
**AGI86379.1**	uncharacterized protein	187	conserved in archaea (DUF531)

Proteins in bold indicate homologs in the two other members of the Methanomassiliicoccales, devoided of Pyl. Proteins in italics indicate homologs in the two other members of the Methanomassiliicoccales also containing Pyl.

^a^Number of amino acids.

Particular genetic signals in the genes containing an in-frame TAG have been proposed to enhance the incorporation of Pyl in the proteins but are not obligatorily requested for that purpose [81]. Two alternative adaptations have been proposed for *Methanosarcina* spp. and the bacteria *Acetohalobium arabaticum* to minimize proteome alteration in consequence of the insertion of Pyl on the stop codons normally intended to stop the translation [77]. In *A. arabaticum* the expression of the Pyl-cassette has been shown to be regulated by substrate (trimethylamine) availability, while in *Methanosarcina* spp. which constitutively express the Pyl-cassette [79, 82], the frequency of genes ended by a TAG stop codon is minimized (~4-5% in *Methanosarcina* spp. *vs*. 20-30% in *A. arabaticum* and other Pyl-decoding bacteria, see Additional file 1: Table S12 adapted from [77]). Accordingly, the extremely low frequency of TAG stop codons in “*Ca.* M. alvus” (1.6%) suggests a constitutive expression of the Pyl-cassette and an efficient ability to incorporate Pyl in proteins (Figure 5, Additional file 1: Table S12). In such tRNA^Pyl^ suppressing context, the apparition of an in-frame *amber* codon in a CDS would lead to a stable mutation as supported by the high occurrence of genes predicted to encode Pyl-containing proteins in “*Ca.* M. alvus”. The phylogenetic position of “*Ca.* M. alvus” among a large cluster of gut methanogens suggests a long evolutionary history in this type of environments where mono- di- and trimethylamine are likely not limiting [17, 83, 84] and may be obtained through the degradation of glycine betaine, choline and L-carnitine by co-occurring microorganisms [85–87]. This high availability of methylamines during the evolution of “*Ca.* M. alvus”, involving a possibly high and constant expression of the Pyl-machinery, could have been a driving factor that has led to this particularly low usage of the triplet TAG in CDSs as termination signals during translation. In addition, the insertion of an *amber* codon in a gene coding for a protein of major function (such as the highly conserved MtxX, Cas1 or the digeranylgeranylglyceryl phosphate synthase in the present case) might have turned the expression of the Pyl cassette and the efficient ability to incorporate Pyl essential for growth. As a feedback this would contribute to tight the association of “*Ca.* M. alvus” cluster methanogens with digestive tract environments. The absence of predicted Pyl-encoding proteins other than MtmB, MtbB and MttB and the high frequency of genes ended by TAG (11.3%) in *M. luminyensis* (Figure 5, Additional file 1: Table S12) argue for a different handling of the Pyl-encoding capacity, possibly through a more important regulation of Pyl-incorporation, and could reflect an adaptation to lower or more variable availability in methylamines [88]. Together with other genomic traits described above, this supports a larger distribution of *M. luminyensis* than digestive tract environments. Following the hypothesis of a methylamine-directed selective pressure on TAG usage in CDSs of the Methanomassiliicoccales, the intermediate TAG usage in the CDSs of “*Ca.* M. intestinalis” (Figure 5, Additional file 1: Table S12) would reflect a more stringent association to digestive tracts compared to *M. luminyensis*.

## Conclusions

Several atypical features were identified in the three genomes such as the scattering of the ribosomal RNA genes and the absence of eukaryotic-like histone gene otherwise present in most of Euryarchaeota genomes. The lack of the eukaryotic-like histone gene could represent an ancestral loss of the overall branch composed by Thermoplasmatales and related lineages, replaced by bacterial-type histone in Thermoplasmatales or Alba protein present in all genomes of the branch with the exception of “*Ca.* M. intestinalis” and “*Ca.* M. alvus”. Intriguingly, the nature of this protein remains elusive in "*Ca.* M. intestinalis" and “*Ca.* M. alvus”.

The absence of a large number of genes otherwise present in all methanogens, but not all restricted to methanogens, was previously reported in *M. luminyensis* and “*Ca.* M. alvus” genomes and is presently extended to “*Ca.* M. intestinalis”. The large lack of these genes involved in the CO_2_ reduction/methyl-oxidation pathways in other methanogens offers a unique context to redefine the genes encoding enzymes or isoenzymes shared by all and only methanogens. Interestingly, the reevaluation shows that this core is not deeply changed when Methanomassiliicoccales are considered. In addition to the genes encoding the Mtr complex, only two of these methanogenesis marker genes are absent from the Methanomassiliicoccales genomes. Gathered with *mcrABG* on a small genomic portion in *M. luminyensis* and “*Ca.* M. alvus”, core genes encoding uncharacterized proteins could be intimately involved in the functioning of the Mcr complex. The process of energy conservation associated to methanogenesis on methyl-compound reduction with H_2_ was analyzed. The original composition of genes presently identified to take part to this process suggests the involvement of a flavin-based electron bifurcation and a membrane associated electron transport chain which are distinctive elements of the two main energy conservation processes defined in other methanogens. However the complete process remains to be uncovered and several components have to be characterized.

While the three Methanomassiliicoccales representatives were cultured from gastrointestinal tract, the analysis of their genome revealed differential adaptations to this environment and possibly contrasted evolutionary history. One of the striking differences among the three species relies on their usage of the TAG codon which could have been shaped by the availability of methylamines as a substrate during their evolution. The long term adaptation of “*Ca.* M. alvus” to GIT environments, suggested by its position among a large cluster of GIT-derived sequences, is supported by its gene composition, along with lateral gene transfer from GIT-associated bacteria. The phylogenetic position of *M. luminyensis* and “*Ca.* M. intestinalis” among soil and sediment methanogens suggests a more recent adaptation or more facultative association to GIT conditions. Consistent with this hypothesis, the *M. luminyensis* genome contains several important genes which are specifically present in soil and sediment methanogens. Although phylogenetically close to *M. luminyensis*, “*Ca.* M. intestinalis” has a reduced genome with a lower [G + C] % and does not share the signatures of soil or sediment adaptations of *M. luminyensis*. These differences could reflect a phenomenon of streamlining in the “*Ca.* M. intestinalis” genome linked with its adaptation to GIT conditions. A similar phenomenon was previously reported from free-living bacteria [29] and with more extreme amplitude, in obligate pathogens [89] as well as in bacterial [90] and archaeal [91] symbionts.

## Methods

### Gene structure prediction

Complete genome sequences of “*Ca.* M. alvus” [GenBank: NC_020913.1] and “*Ca.* M. intestinalis” [GenBank: NC_021353.1] were obtained from enriched consortia of stool-derived cultures from a 91-year-old woman, with an average genome sequence coverage respectively of 36.9 fold and 42.7 fold [7, 8]. Genomic sequences from *Methanomassiliicoccus luminyensis* B10 were retrieved from the Genbank database [GenBank: CAJE01000001-CAJE01000026]. Raw sequences from “*Ca.* M. alvus” Mx1201, “*Ca.* M. intestinalis” Mx1-Issoire and *M. luminyensis* were fed to the RAST Annotation server [92] using Glimmer3 [93] for open-reading frames prediction. The RAST Annotation used the released 59 of FIGfam and no frameshifts fixing parameters. To perform an accurate structural annotation of these genomes, a comparative analysis of the “*Ca.* M. alvus”, “*Ca.* M. intestinalis” and *M. luminyensis* annotated proteomes was conducted using the TBLASTN program. To identify genes or distantly related genes, a BLOSUM45 substitution matrix was chosen, and low-complexity filters were suppressed. TBLASTN analyses were manually validated to take into account genes with frame-shifts due to sequencing errors. Translation start codons were then validated through a BLASTP comparative analysis of the three annotated proteomes. Protein sequences from the three proteomes were compared together with the curated SWISS-PROT protein sequences database [94]. Results were filtered using 80% length and 40% identity thresholds and start codons were manually corrected taking into account protein sizes and local alignments. Non-coding RNAs were predicted using the Rfam database [95] with an E-value threshold of 1 and results were manually curated. Additional analyses were performed to detect tRNAs by merging results from tRNAscan [96], TFAM [97], ARAGORN [98] and BLASTN [99]. CRISPRFinder [100] was applied for each of the three genomes to detect CRISPR loci that were compared together using CRISPRcompar [101] and CRISPRmap [102]. Finally, prophages were sought using PHAST [103]. Circular representation of the “*Ca.* M. alvus” and “*Ca.* M. intestinalis” genomes were performed using the CGView Server [104].

### Comparative genome analysis and functional annotation

An ‘all-*versus*-all’ BLASTP comparison of the predicted protein sequences within each of the three genomes was conducted [99]. On the basis of the best BLASTP hits, orthologous relationships were established between the protein sequences of “*Ca.* M. alvus”, “*Ca.* M. intestinalis” and *M. luminyensis*. A Venn diagram was then drawn using the Venny web service [105]. Predicted functions provided by the RAST annotation server for each CDS of the three species were kept as functional annotation. Using orthology relationships previously established, a functional annotation transfer was performed. Protein sequences of genes with frame-shift mutations were manually reconstructed. In order to distinguish protein sequences only found within the three genomes and shared protein sequences with closely related species, a BLASTP analysis was conducted. Each protein sequence from the core proteome was compared to i) phylogenetic neighbors proteomes (*Aciduliprofundum boonei* T469, accession code: NC_013926; *Aciduliprofundum sp.* MAR08-339, accession code: NC_019942; *Ferroplasma acidarmanus* fer1, accession code: CM000428; *Thermoplasma acidophilum* DSM 1728, accession code: NC_002578; *Thermoplasma volcanium* DSS1, accession code: NC_002689 and MG-II, accession code: CM001443), ii) methanogenic archaeon from human gut (*Methanobrevibacter smithii* ATCC 35061, accession code: NC_009515 and *Methanosphaera stadtmanae* DSM 3091, accession code: NC_007681) and iii) the NCBI non-redundant protein sequences database (release 12/2012). Identity threshold was set at 30% with a minimum length coverage of 80%. An arCOG [45] analysis was also performed using the December 2012 release (ftp://ftp.ncbi.nih.gov/pub/wolf/COGs/arCOG/). Each annotated protein sequence from the three genomes was compared to the arCOG database using BLASTP and an E-value threshold equal to 1e^-3^. The arCOG profiles of the three genomes and those of the arCOG database were used to identify proteins potentially shared by all and only methanogens, as well as proteins almost specific to methanogens and shared by almost all methanogens. Distribution of each selected protein among sequenced organisms was checked by BLASTP. Conserved domains of the selected proteins were compared to those of the closest results that belong to non-methanogens and phylogenetic three were constructed to verify their monophyly. Additional proteomes from various archaeal orders were also submitted to this comparison: *A. boonei* T469; *Archaeoglobus fulgidus* DSM 4304, accession code: NC_013926; *Archaeoglobus veneficus* SNP6, accession code: NC_015320; *M. smithii* ATCC 35061; *M. stadtmanae* DSM 3091; *Thermoplasma acidophilum* DSM 1728, accession code: NC_002578 and MG-II. In order to detect putative lateral gene transfers, the same BLASTP analysis was performed for the three proteomes using the UniprotKB database [106]. Only best hits were retrieved and classified according to the three domains of life: Archaea, Bacteria or Eukaryota. The genomes of the Methanomassiliicoccales representatives were not included in the subject database. Metabolic pathways reconstruction was performed through the KEGG Automatic Annotation Server (KAAS) [107] using a bi-directional best hit strategy and a custom list of reference organisms. Indeed, based on best BLAST hit results from the three proteomes, 40 species were selected for the KAAS (three-letter organism codes are listed as follows: abi, mac, tac, mba, rci, mig, afu, mpd, tba, mpi, pab, mka, pho, mhu, mja, mla, mth, cdc, amt, drm, mbn, ssg, ele, fnu, mel, mrv, fsv, tsi, lba, ral, sti, msi, sce, eco, ere, aas, eha, sfu, bla, cau). The transportome was determined using the TransportTP server [108] (reference organism: *Escherichia coli*; E-value threshold: 0.1). Results were manually validated and curated using BLASTP analysis using transportDB [109] and taking into account orthology relationships. Signal peptides, transmembrane helices and PFAM domains were predicted through the InterProScan annotation module provided by the BLAST2GO software [110] with default parameters.

### Phylogenomic analysis of DNA replication components

Homologs of each major archaeal DNA replication component were retrieved from the reference sequence database at the NCBI using the BLASTP program with different seeds from each archaeal order [99]. The top 100 best hits for each order were then used to create HMM profiles [111] (http://www.hmmer.org) that allowed iteratively searching a local database of 142 complete or nearly complete archaeal genomes including 98 plasmid sequences, as well as in a local database of the available complete archaeal virus genomes (56 total) downloaded from the Viral Genomes database of NCBI (as of June 20^th^ 2013). Absences of a given homolog in a specific genome were verified by performing additional TBLASTN searches [99]. Multiple alignments were done with MUSCLE 3.8.31 [112] and manually inspected using the ED program from the MUST package to remove non-homologous or partial sequences [113]. The alignments were trimmed using the software BMGE [114] with default parameters. Phylogenetic analyses were performed on single protein datasets using Maximum Likelihood and Bayesian methods. Maximum likelihood analyses were performed with RaxML [115]. Mr. Bayes 3.2 [116] was used to perform Bayesian analyses using the mixed amino acid substitution model and four categories of evolutionary rates. Two independent runs were performed for each data set, and runs were stopped when they reached a standard deviation of split frequency below 0.01 or the log likelihood values reached stationarity. The majority-rule consensus trees were obtained after discarding first 25% samples as ‘burn-in’.

### Data access

The whole genome shotgun projects, the complete genome sequences and annotations have been deposited at DDBJ/EMBL/GenBank for “*Ca.* M. alvus” Mx1201 [GenBank: CP004049] and for “*Ca.* M. intestinalis” Issoire-Mx1 [GenBank: CP005934]. Predicted CDS and protein sequences for *M. luminyensis*, some of which are not annotated in GenBank are provided respectively through Additional file 1: Tables S12 and S13.

## Electronic supplementary material

Additional file 1: **Additional tables in a zipped folder containing:** **Table S1.** tRNA and ncRNA contents for the genomes of the three Methanomassiliicoccales representatives. **Table S2.** Codon usage in the three genomes of Methanomassiliicoccales. **Table S3.** CRISPR DR elements found in the three genomes. **Table S4.** Number of best hits score among the three domains of life. **Table S5.** Genes list of the core genome of the Methanomassiliicoccales, as deduced by a TBLASTN analysis (with reference to CDS of “*Ca*. M. alvus” genome), and their presence or not in phylogenetical neighbors, human gut Methanobacteriales and non-redundant genbank DB. **Table S6.** CDS list of the core genome of the Methanomassiliicoccales, absent in phylogenetical neighbors and the human gut Methanobacteriales. In blue, the 20 CDS not retrieved in genbank database. **Table S7.** arCOG distribution among the Methanomassiliicoccales representative genomes, gut methanogens and some other archaea. **Table S8.** Complete list of transporters detected by TransportDB, in the three genomes of Methanomassiliicoccales. **Table S9.** List of the antioxydant systems in the three genomes of Methanomassiliicoccales. **Table S10.** Genes involved in methanogenesis in "*Ca*. M. alvus", "*Ca*. M. intestinalis" and *M. luminyensis* and accession numbers of the proteins they encode. **Table S11.** Comparative presence of the genes involved in the synthesis of the coM among the seven orders of methanogens. **Table S12.** Numbers of CDS with in-frame TAG, and % of the total CDS in various genomes of microorganisms coding or not pyrrolysine (update information from Prat *et al*. [77]). **Table S13.** CDS list of *M. luminyensis* B10. **Table S14.** Proteome of *M. luminyensis* B10. (ZIP 1 MB)

Additional file 2: **Additional figures in a zipped folder containing:** **Figure S1.** CRISPR Direct Repeats structure. The figure shows the 2D, Minimum Free Energy structure of CRISPR DRs retrieved from the three genomes of the Methanomassiliicoccales (using RNAfold web server [117]) and the sequence alignment of *M. luminyensis* DR with the family 3, motif 27 DRs (using CRISPRmap [34])**. Figure S2.** Chromosome circular maps of (A) “*Candidatus* Methanomethylophilus alvus” Mx1201 and (B) “*Candidatus* Methanomassiliicoccus intestinalis” Mx1-Issoire genomes (generated with CGView [104]). Circles display from outside: 1 and 4, rRNA genes respectively on forward and reverse strand; 2 and 3, CDS on forward and reverse strand; 5, BLASTX results with a maximum expected value of 1e^-3^ *versus* the “*Ca.* M. intestinalis” proteome; 6, [G + C] % content deviation from the average [G + C] % content of the genome. Arrows, location and sense of the *orc1/cdc6* genes. **Figure S3.** Phylogeny of Cdc6/Orc1 proteins. **Figure S4.** Phylogenetic trees of NAD-dependent DNA ligase (A) and Choloyglycine hydrolase (B) genes likely transferred from bacteria to "*Ca.* M. alvus". In red, sequences of "*Ca.* M. alvus", in blue sequences from other gut-associated methanogens. **Figure S5.** Metabolic comparison of the three genomes based on KEGG maps. Series of three boxes represent presence or absence of the E.C. numbered enzyme (yellow for “*Ca.* M. alvus”, green for “*Ca.* M. intestinalis” and blue for *M. luminyensis*). Green arrows replace complex pathways. Blue boxes, synthetized compounds by the 3 species; Red boxes, compounds not synthetized by the three species. Orange boxes, compounds synthetized by at least 1 species. Question marks show pathways where there is at least one enzyme missing. **Figure S6.** Comparison of the physical map of genes involved in methanogenesis on methyl compounds + H_2_ in the three analyzed genomes. (ZIP 4 MB)

Additional file 3: **Additional Data in a zipped MS Word file.** Details on lipids, amino acids and purine synthesis, as well as molecular nitrogen fixation deduced from the genomes of the three members of the Methanomassiliicoccales. (ZIP 26 KB)

## Abbreviations

aaRS: Aminoacyl tRNA synthetase
AIR: 5-amino -4-imidazole ribonucleotide
arCOG: Archaeal Cluster of Orthologous Genes
ASAT: Aspartate aminotransferase
BSH: Bile Salt Hydrolase
CAIR: 5-amino-4-imidazole carboxylic acid ribonucleotide
Cdc6: Cell division cycle 6
CDS: Coding DNA sequence
CRISPR: Clustered Regularly Interspaced Short Palindromic Repeat
CS: Cysteate Synthase
DHNOPT: 1,4-dihydroxy-2-naphthoate octaprenyltransferase
DMS: Dimethylsulfide
DPL: DNA polymerase D large subunit
DPM: Dolichol-phosphate mannose
DPS: DNA polymerase D small subunit
FEN-1: Flap EndoNuclease 1
GGPS: (S)-3-O-geranylgeranylglyceryl phosphate synthase
GINS: Go-Ichi-Nii-San protein
GIT: Gastro-intestinal tract
GT: Glycosyl Transferase
H_4_MP: Tetrahydromethanopterin
Hec: Unknown hydrogenase, probable energy-converting
HPNQMT: 2-heptaprenyl-1,4-naphthoquinone methyltransferase
IPPK: Isopentenyl phosphate kinase
LGT: Lateral Gene Transfer
LSU: Large subunit
MCM: Minichromosome maintenance protein
MG-II: Uncultured Marine Group II
NCAIR: N5-5-amino -4-imidazole carboxylic acid ribonucleotide
nr: Non-redundant
ORB: Origin recognition box
ORF: Open Reading Frame
Ori: Origin of replication
PCNA: Proliferating Cell Nuclear Antigen
PFOR: Pyruvate:ferredoxin oxydoreductase
PMDC: Phosphomevalonate decarboxylase
PMK: Phosphomevalonate kinase
PPS: Polyprenyl synthetase
PriL: Primase large subunit
PriS: Primase small subunit
PRPP: Phosphoribosyl pyrophosphate
Pyl: Pyrrolysine
RCC: Rumen cluster C
RFC: Replication Factor C
RNaseH: Ribonuclease H
RPA: Replicative Protein A
SD: Standard Deviation
SSB: Single Strand Binding protein
SSU: small subunit
TMA: Trimethylamine
Topo: Topoisomerase.
