# Supplementary material for: Comparative genomics highlights the unique biology of Methanomassiliicoccales, a Thermoplasmatales-related seventh order of methanogenic archaea that encodes pyrrolysine
Source: BMC Genomics. 2014 Aug 13;15:679. doi: 10.1186/1471-2164-15-679 (PMC4153887; doi:10.1186/1471-2164-15-679)
Supplement: Supplementary file 1 — Additional file 1: Additional tables in a zipped folder containing: Table S1. tRNA and ncRNA contents for the genomes of the three Methanomassiliicoccales representatives. Table S2. Codon usage in the three genomes of Methanomassiliicoccales. Table S3. CRISPR DR elements found in the three genomes. Table S4. Number of best hits score among the three domains of life. Table S5. Genes list of the core genome of the Methanomassiliicoccales, as deduced by a TBLASTN analysis (with reference to CDS of “Ca. M. alvus” genome), and their presence or not in phylogenetical neighbors, human gut Methanobacteriales and non-redundant genbank DB. Table S6. CDS list of the core genome of the Methanomassiliicoccales, absent in phylogenetical neighbors and the human gut Methanobacteriales. In blue, the 20 CDS not retrieved in genbank database. Table S7. arCOG distribution among the Methanomassiliicoccales representative genomes, gut methanogens and some other archaea. Table S8. Complete list of transporters detected by TransportDB, in the three genomes of Methanomassiliicoccales. Table S9. List of the antioxydant systems in the three genomes of Methanomassiliicoccales. Table S10. Genes involved in methanogenesis in "Ca. M. alvus", "Ca. M. intestinalis" and M. luminyensis and accession numbers of the proteins they encode. Table S11. Comparative presence of the genes involved in the synthesis of the coM among the seven orders of methanogens. Table S12. Numbers of CDS with in-frame TAG, and % of the total CDS in various genomes of microorganisms coding or not pyrrolysine (update information from Prat et al. [77]). Table S13. CDS list of M. luminyensis B10. Table S14. Proteome of M. luminyensis B10. (ZIP 1 MB) [file 12864_2014_6390_MOESM1_ESM.zip › 2014_BMCGenomics_Additional_Table S4_Best Hits among domains.docx]

**Additional Table S4**

**Number of best hits score among the three domains of life**

|  | Archaea | Bacteria | Eukaryota | # hits |
| --- | --- | --- | --- | --- |
| "*Ca*. M. alvus" | 980 (69.7%) | 424 (30.1%) | 2 (0.2%) | 1406 |
| "*Ca*. M. intestinalis" | 1292 (79.3%) | 333 (20.4%) | 5 (0.3%) | 1630 |
| *M. luminyensis* | 1817 (81.7%) | 403 (18.1%) | 4 (0.2%) | 2224 |

A BLASTP analysis was performed for each proteome of the three members of the 7^th^ methanogenic order, against the UNIPROTKB database using an E-value threshold of 1e-3.
