# Supplementary material for: Comparative genomics highlights the unique biology of Methanomassiliicoccales, a Thermoplasmatales-related seventh order of methanogenic archaea that encodes pyrrolysine
Source: BMC Genomics. 2014 Aug 13;15:679. doi: 10.1186/1471-2164-15-679 (PMC4153887; doi:10.1186/1471-2164-15-679)
Supplement: Supplementary file 2 — Additional file 2: Additional figures in a zipped folder containing: Figure S1. CRISPR Direct Repeats structure. The figure shows the 2D, Minimum Free Energy structure of CRISPR DRs retrieved from the three genomes of the Methanomassiliicoccales (using RNAfold web server [117]) and the sequence alignment of M. luminyensis DR with the family 3, motif 27 DRs (using CRISPRmap [34]). Figure S2. Chromosome circular maps of (A) “Candidatus Methanomethylophilus alvus” Mx1201 and (B) “Candidatus Methanomassiliicoccus intestinalis” Mx1-Issoire genomes (generated with CGView [104]). Circles display from outside: 1 and 4, rRNA genes respectively on forward and reverse strand; 2 and 3, CDS on forward and reverse strand; 5, BLASTX results with a maximum expected value of 1e-3 versus the “Ca. M. intestinalis” proteome; 6, [G + C] % content deviation from the average [G + C] % content of the genome. Arrows, location and sense of the orc1/cdc6 genes. Figure S3. Phylogeny of Cdc6/Orc1 proteins. Figure S4. Phylogenetic trees of NAD-dependent DNA ligase (A) and Choloyglycine hydrolase (B) genes likely transferred from bacteria to "Ca. M. alvus". In red, sequences of "Ca. M. alvus", in blue sequences from other gut-associated methanogens. Figure S5. Metabolic comparison of the three genomes based on KEGG maps. Series of three boxes represent presence or absence of the E.C. numbered enzyme (yellow for “Ca. M. alvus”, green for “Ca. M. intestinalis” and blue for M. luminyensis). Green arrows replace complex pathways. Blue boxes, synthetized compounds by the 3 species; Red boxes, compounds not synthetized by the three species. Orange boxes, compounds synthetized by at least 1 species. Question marks show pathways where there is at least one enzyme missing. Figure S6. Comparison of the physical map of genes involved in methanogenesis on methyl compounds + H2 in the three analyzed genomes. (ZIP 4 MB) [file 12864_2014_6390_MOESM2_ESM.zip › 2014_BMCGenomics_Additional_Figure S2_circular maps.pptx]

## Slide 1
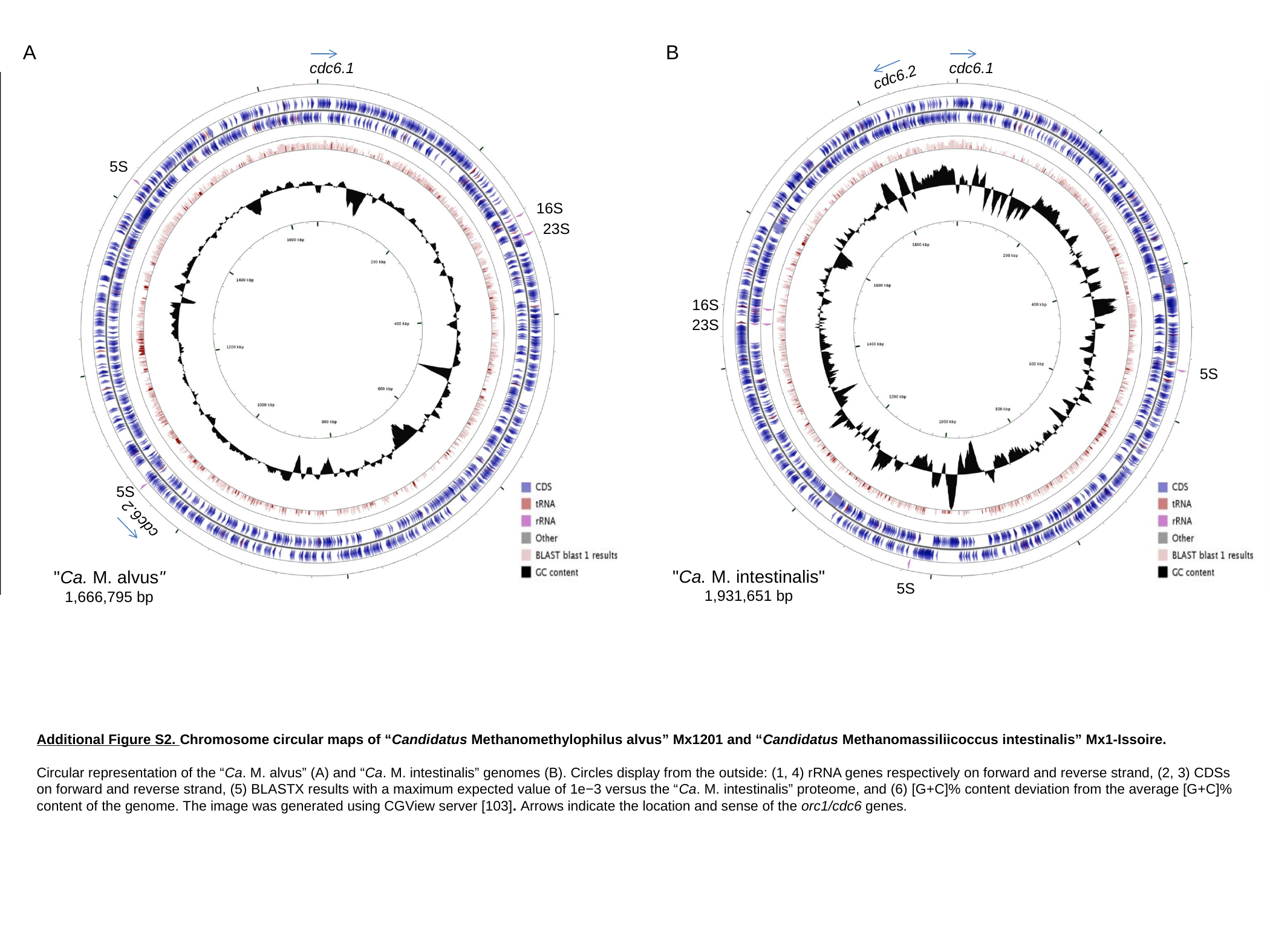

A
cdc6.1
5S
16S
23S
5S
cdc6.2
"Ca. M. alvus"
1,666,795 bp
B
cdc6.1
cdc6.2
16S
23S
5S
"Ca. M. intestinalis"
1,931,651 bp
5S
Additional Figure S2. Chromosome circular maps of “Candidatus Methanomethylophilus alvus” Mx1201 and “Candidatus Methanomassiliicoccus intestinalis” Mx1-Issoire.
Circular representation of the “Ca. M. alvus” (A) and “Ca. M. intestinalis” genomes (B). Circles display from the outside: (1, 4) rRNA genes respectively on forward and reverse strand, (2, 3) CDSs on forward and reverse strand, (5) BLASTX results with a maximum expected value of 1e−3 versus the “Ca. M. intestinalis” proteome, and (6) [G+C]% content deviation from the average [G+C]% content of the genome. The image was generated using CGView server [103]. Arrows indicate the location and sense of the orc1/cdc6 genes.
