# Supplementary material for: Comparative genomics highlights the unique biology of Methanomassiliicoccales, a Thermoplasmatales-related seventh order of methanogenic archaea that encodes pyrrolysine
Source: BMC Genomics. 2014 Aug 13;15:679. doi: 10.1186/1471-2164-15-679 (PMC4153887; doi:10.1186/1471-2164-15-679)
Supplement: Supplementary file 2 — Additional file 2: Additional figures in a zipped folder containing: Figure S1. CRISPR Direct Repeats structure. The figure shows the 2D, Minimum Free Energy structure of CRISPR DRs retrieved from the three genomes of the Methanomassiliicoccales (using RNAfold web server [117]) and the sequence alignment of M. luminyensis DR with the family 3, motif 27 DRs (using CRISPRmap [34]). Figure S2. Chromosome circular maps of (A) “Candidatus Methanomethylophilus alvus” Mx1201 and (B) “Candidatus Methanomassiliicoccus intestinalis” Mx1-Issoire genomes (generated with CGView [104]). Circles display from outside: 1 and 4, rRNA genes respectively on forward and reverse strand; 2 and 3, CDS on forward and reverse strand; 5, BLASTX results with a maximum expected value of 1e-3 versus the “Ca. M. intestinalis” proteome; 6, [G + C] % content deviation from the average [G + C] % content of the genome. Arrows, location and sense of the orc1/cdc6 genes. Figure S3. Phylogeny of Cdc6/Orc1 proteins. Figure S4. Phylogenetic trees of NAD-dependent DNA ligase (A) and Choloyglycine hydrolase (B) genes likely transferred from bacteria to "Ca. M. alvus". In red, sequences of "Ca. M. alvus", in blue sequences from other gut-associated methanogens. Figure S5. Metabolic comparison of the three genomes based on KEGG maps. Series of three boxes represent presence or absence of the E.C. numbered enzyme (yellow for “Ca. M. alvus”, green for “Ca. M. intestinalis” and blue for M. luminyensis). Green arrows replace complex pathways. Blue boxes, synthetized compounds by the 3 species; Red boxes, compounds not synthetized by the three species. Orange boxes, compounds synthetized by at least 1 species. Question marks show pathways where there is at least one enzyme missing. Figure S6. Comparison of the physical map of genes involved in methanogenesis on methyl compounds + H2 in the three analyzed genomes. (ZIP 4 MB) [file 12864_2014_6390_MOESM2_ESM.zip › 2014_BMCGenomics_Additional_Figure S4_Phylogenetic threes of predicted LGT NAD-dependent DNA ligase and Choloyglycine hydrolase gene in Ca M. alvus.pptx]

## Slide 1
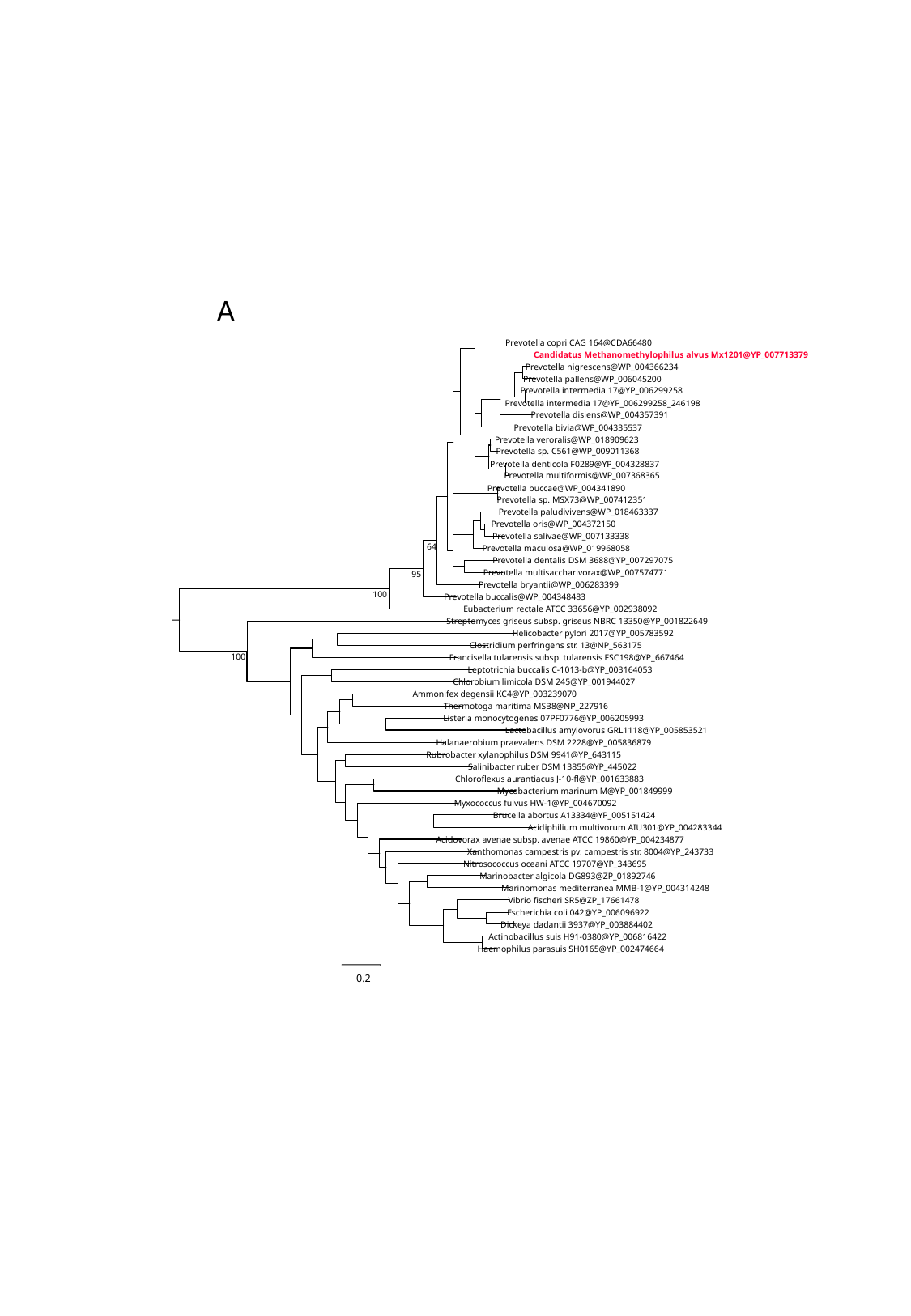

A
Prevotella copri CAG 164@CDA66480
Candidatus Methanomethylophilus alvus Mx1201@YP_007713379
Prevotella nigrescens@WP_004366234
Prevotella pallens@WP_006045200
Prevotella intermedia 17@YP_006299258
Prevotella intermedia 17@YP_006299258_246198
Prevotella disiens@WP_004357391
Prevotella bivia@WP_004335537
Prevotella veroralis@WP_018909623
Prevotella sp. C561@WP_009011368
Prevotella denticola F0289@YP_004328837
Prevotella multiformis@WP_007368365
Prevotella buccae@WP_004341890
Prevotella sp. MSX73@WP_007412351
Prevotella paludivivens@WP_018463337
Prevotella oris@WP_004372150
Prevotella salivae@WP_007133338
64
Prevotella maculosa@WP_019968058
Prevotella dentalis DSM 3688@YP_007297075
Prevotella multisaccharivorax@WP_007574771
95
Prevotella bryantii@WP_006283399
100
Prevotella buccalis@WP_004348483
Eubacterium rectale ATCC 33656@YP_002938092
Streptomyces griseus subsp. griseus NBRC 13350@YP_001822649
Helicobacter pylori 2017@YP_005783592
Clostridium perfringens str. 13@NP_563175
100
Francisella tularensis subsp. tularensis FSC198@YP_667464
Leptotrichia buccalis C-1013-b@YP_003164053
Chlorobium limicola DSM 245@YP_001944027
Ammonifex degensii KC4@YP_003239070
Thermotoga maritima MSB8@NP_227916
Listeria monocytogenes 07PF0776@YP_006205993
Lactobacillus amylovorus GRL1118@YP_005853521
Halanaerobium praevalens DSM 2228@YP_005836879
Rubrobacter xylanophilus DSM 9941@YP_643115
Salinibacter ruber DSM 13855@YP_445022
Chloroflexus aurantiacus J-10-fl@YP_001633883
Mycobacterium marinum M@YP_001849999
Myxococcus fulvus HW-1@YP_004670092
Brucella abortus A13334@YP_005151424
Acidiphilium multivorum AIU301@YP_004283344
Acidovorax avenae subsp. avenae ATCC 19860@YP_004234877
Xanthomonas campestris pv. campestris str. 8004@YP_243733
Nitrosococcus oceani ATCC 19707@YP_343695
Marinobacter algicola DG893@ZP_01892746
Marinomonas mediterranea MMB-1@YP_004314248
Vibrio fischeri SR5@ZP_17661478
Escherichia coli 042@YP_006096922
Dickeya dadantii 3937@YP_003884402
Actinobacillus suis H91-0380@YP_006816422
Haemophilus parasuis SH0165@YP_002474664
0.2

## Slide 2
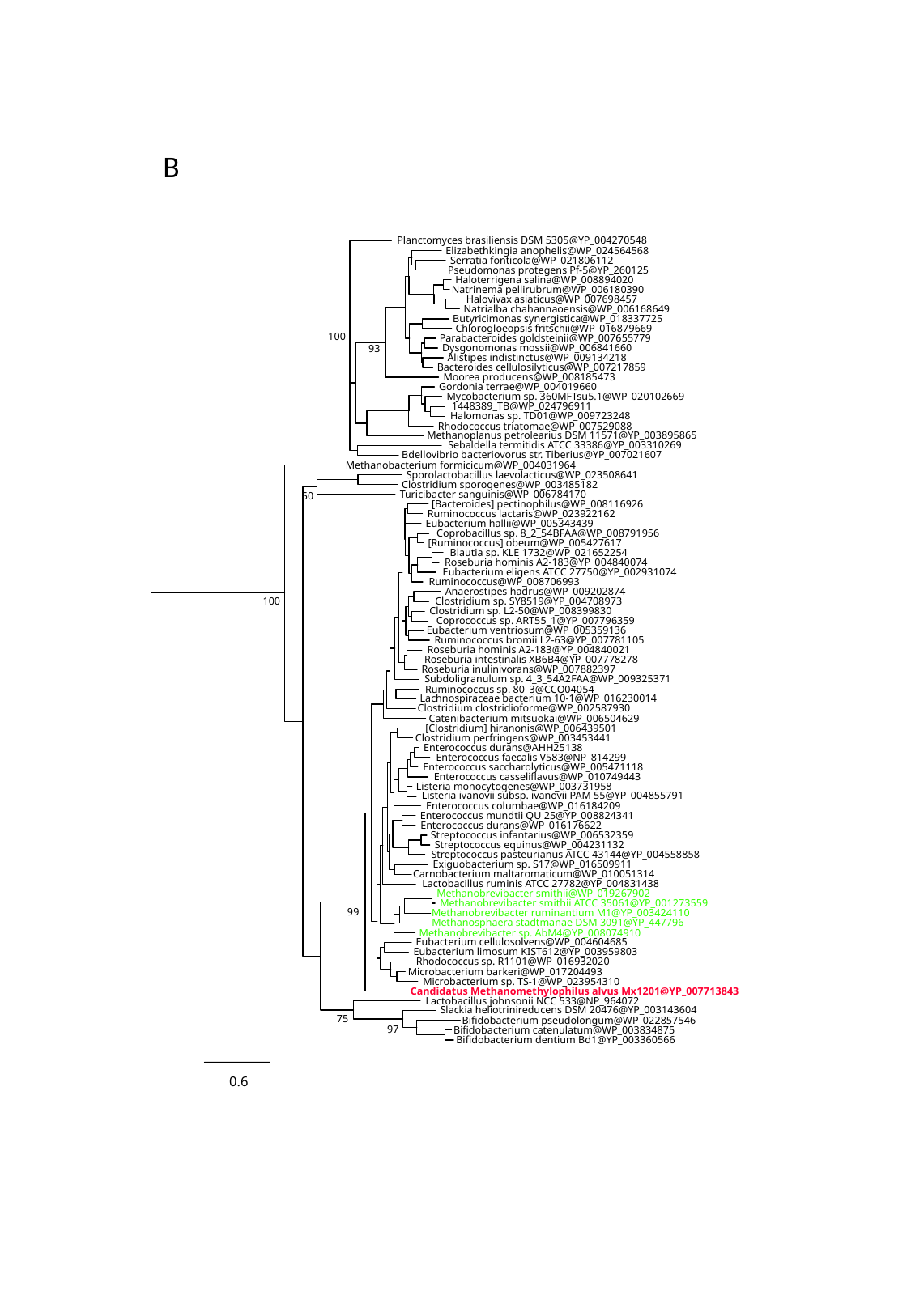

B
Planctomyces brasiliensis DSM 5305@YP_004270548
Elizabethkingia anophelis@WP_024564568
Serratia fonticola@WP_021806112
Pseudomonas protegens Pf-5@YP_260125
Haloterrigena salina@WP_008894020
Natrinema pellirubrum@WP_006180390
Halovivax asiaticus@WP_007698457
Natrialba chahannaoensis@WP_006168649
Butyricimonas synergistica@WP_018337725
Chlorogloeopsis fritschii@WP_016879669
100
Parabacteroides goldsteinii@WP_007655779
Dysgonomonas mossii@WP_006841660
93
Alistipes indistinctus@WP_009134218
Bacteroides cellulosilyticus@WP_007217859
Moorea producens@WP_008185473
Gordonia terrae@WP_004019660
Mycobacterium sp. 360MFTsu5.1@WP_020102669
1448389_TB@WP_024796911
Halomonas sp. TD01@WP_009723248
Rhodococcus triatomae@WP_007529088
Methanoplanus petrolearius DSM 11571@YP_003895865
Sebaldella termitidis ATCC 33386@YP_003310269
Bdellovibrio bacteriovorus str. Tiberius@YP_007021607
Methanobacterium formicicum@WP_004031964
Sporolactobacillus laevolacticus@WP_023508641
Clostridium sporogenes@WP_003485182
Turicibacter sanguinis@WP_006784170
50
[Bacteroides] pectinophilus@WP_008116926
Ruminococcus lactaris@WP_023922162
Eubacterium hallii@WP_005343439
Coprobacillus sp. 8_2_54BFAA@WP_008791956
[Ruminococcus] obeum@WP_005427617
Blautia sp. KLE 1732@WP_021652254
Roseburia hominis A2-183@YP_004840074
Eubacterium eligens ATCC 27750@YP_002931074
Ruminococcus@WP_008706993
Anaerostipes hadrus@WP_009202874
100
Clostridium sp. SY8519@YP_004708973
Clostridium sp. L2-50@WP_008399830
Coprococcus sp. ART55_1@YP_007796359
Eubacterium ventriosum@WP_005359136
Ruminococcus bromii L2-63@YP_007781105
Roseburia hominis A2-183@YP_004840021
Roseburia intestinalis XB6B4@YP_007778278
Roseburia inulinivorans@WP_007882397
Subdoligranulum sp. 4_3_54A2FAA@WP_009325371
Ruminococcus sp. 80_3@CCO04054
Lachnospiraceae bacterium 10-1@WP_016230014
Clostridium clostridioforme@WP_002587930
Catenibacterium mitsuokai@WP_006504629
[Clostridium] hiranonis@WP_006439501
Clostridium perfringens@WP_003453441
Enterococcus durans@AHH25138
Enterococcus faecalis V583@NP_814299
Enterococcus saccharolyticus@WP_005471118
Enterococcus casseliflavus@WP_010749443
Listeria monocytogenes@WP_003731958
Listeria ivanovii subsp. ivanovii PAM 55@YP_004855791
Enterococcus columbae@WP_016184209
Enterococcus mundtii QU 25@YP_008824341
Enterococcus durans@WP_016176622
Streptococcus infantarius@WP_006532359
Streptococcus equinus@WP_004231132
Streptococcus pasteurianus ATCC 43144@YP_004558858
Exiguobacterium sp. S17@WP_016509911
Carnobacterium maltaromaticum@WP_010051314
Lactobacillus ruminis ATCC 27782@YP_004831438
Methanobrevibacter smithii@WP_019267902
Methanobrevibacter smithii ATCC 35061@YP_001273559
99
Methanobrevibacter ruminantium M1@YP_003424110
Methanosphaera stadtmanae DSM 3091@YP_447796
Methanobrevibacter sp. AbM4@YP_008074910
Eubacterium cellulosolvens@WP_004604685
Eubacterium limosum KIST612@YP_003959803
Rhodococcus sp. R1101@WP_016932020
Microbacterium barkeri@WP_017204493
Microbacterium sp. TS-1@WP_023954310
Candidatus Methanomethylophilus alvus Mx1201@YP_007713843
Lactobacillus johnsonii NCC 533@NP_964072
Slackia heliotrinireducens DSM 20476@YP_003143604
75
Bifidobacterium pseudolongum@WP_022857546
97
Bifidobacterium catenulatum@WP_003834875
Bifidobacterium dentium Bd1@YP_003360566
0.6
