# Supplementary material for: Comparative genomics highlights the unique biology of Methanomassiliicoccales, a Thermoplasmatales-related seventh order of methanogenic archaea that encodes pyrrolysine
Source: BMC Genomics. 2014 Aug 13;15:679. doi: 10.1186/1471-2164-15-679 (PMC4153887; doi:10.1186/1471-2164-15-679)
Supplement: Supplementary file 2 — Additional file 2: Additional figures in a zipped folder containing: Figure S1. CRISPR Direct Repeats structure. The figure shows the 2D, Minimum Free Energy structure of CRISPR DRs retrieved from the three genomes of the Methanomassiliicoccales (using RNAfold web server [117]) and the sequence alignment of M. luminyensis DR with the family 3, motif 27 DRs (using CRISPRmap [34]). Figure S2. Chromosome circular maps of (A) “Candidatus Methanomethylophilus alvus” Mx1201 and (B) “Candidatus Methanomassiliicoccus intestinalis” Mx1-Issoire genomes (generated with CGView [104]). Circles display from outside: 1 and 4, rRNA genes respectively on forward and reverse strand; 2 and 3, CDS on forward and reverse strand; 5, BLASTX results with a maximum expected value of 1e-3 versus the “Ca. M. intestinalis” proteome; 6, [G + C] % content deviation from the average [G + C] % content of the genome. Arrows, location and sense of the orc1/cdc6 genes. Figure S3. Phylogeny of Cdc6/Orc1 proteins. Figure S4. Phylogenetic trees of NAD-dependent DNA ligase (A) and Choloyglycine hydrolase (B) genes likely transferred from bacteria to "Ca. M. alvus". In red, sequences of "Ca. M. alvus", in blue sequences from other gut-associated methanogens. Figure S5. Metabolic comparison of the three genomes based on KEGG maps. Series of three boxes represent presence or absence of the E.C. numbered enzyme (yellow for “Ca. M. alvus”, green for “Ca. M. intestinalis” and blue for M. luminyensis). Green arrows replace complex pathways. Blue boxes, synthetized compounds by the 3 species; Red boxes, compounds not synthetized by the three species. Orange boxes, compounds synthetized by at least 1 species. Question marks show pathways where there is at least one enzyme missing. Figure S6. Comparison of the physical map of genes involved in methanogenesis on methyl compounds + H2 in the three analyzed genomes. (ZIP 4 MB) [file 12864_2014_6390_MOESM2_ESM.zip › 2014_BMCGenomics_Additional_Figure S6_GenesMethanogenesis.pptx]

## Slide 1
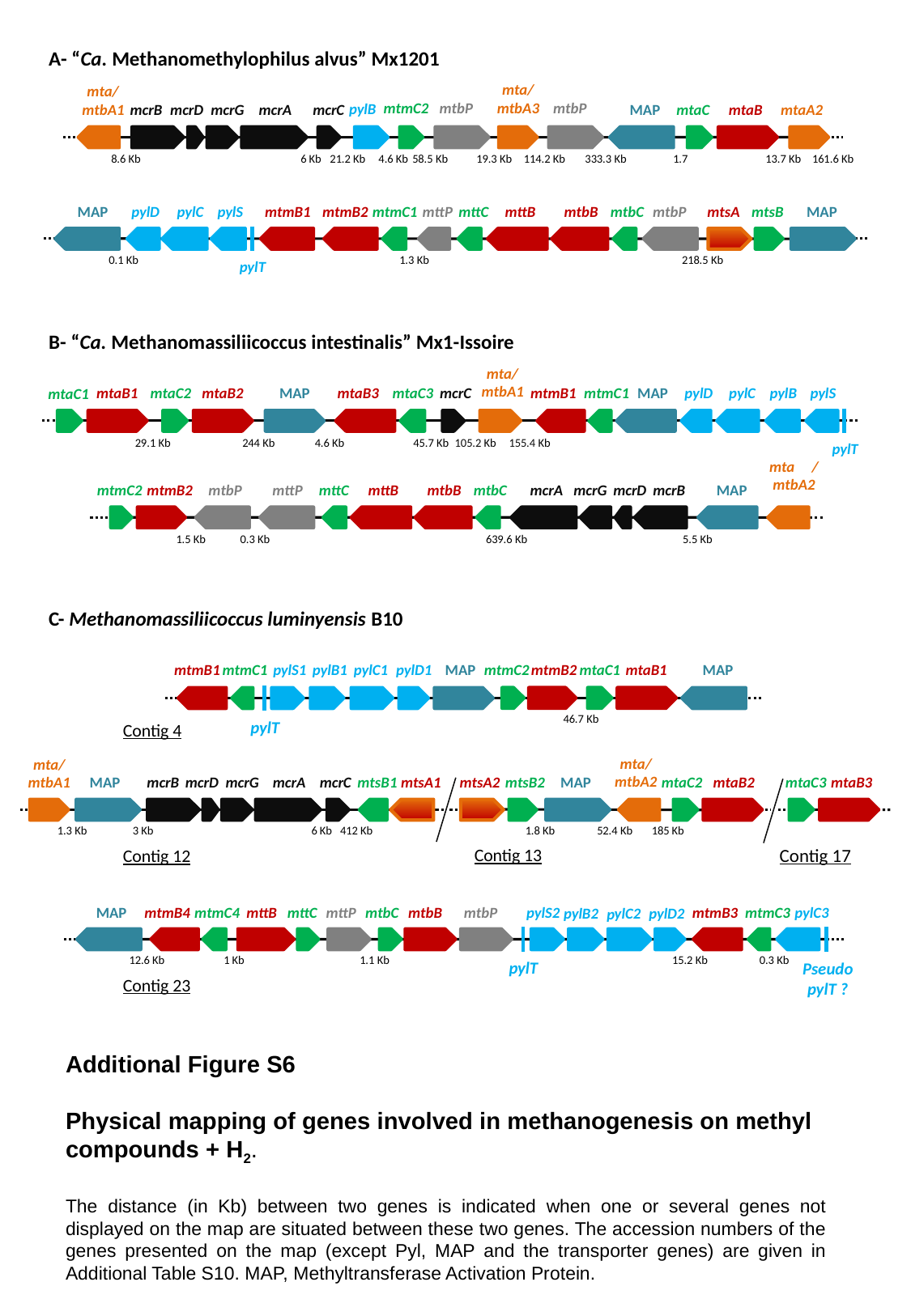

A- “Ca. Methanomethylophilus alvus” Mx1201
mta/ mtbA3
mta/ mtbA1
mtmC2
mtbP
mtbP
pylB
mcrB
mcrD
mcrG
mcrA
mcrC
MAP
mtaC
mtaB
mtaA2
8.6 Kb
6 Kb
21.2 Kb
4.6 Kb
58.5 Kb
19.3 Kb
114.2 Kb
333.3 Kb
1.7
13.7 Kb
161.6 Kb
MAP
pylD
pylC
pylS
mtmB1
mtmB2
mtmC1
mttP
mttC
mttB
mtbB
mtbC
mtbP
mtsA
mtsB
MAP
0.1 Kb
1.3 Kb
218.5 Kb
pylT
B- “Ca. Methanomassiliicoccus intestinalis” Mx1-Issoire
mta/ mtbA1
mtaB1
mtaC2
mtaB2
MAP
mtaB3
mtaC3
mcrC
mtmB1
mtmC1
MAP
pylD
pylC
pylB
pylS
mtaC1
29.1 Kb
244 Kb
4.6 Kb
45.7 Kb
105.2 Kb
155.4 Kb
pylT
mta /mtbA2
mtmC2
mtmB2
mtbP
mttP
mttC
mttB
mtbB
mtbC
MAP
mcrB
mcrA
mcrG
mcrD
1.5 Kb
0.3 Kb
639.6 Kb
5.5 Kb
C- Methanomassiliicoccus luminyensis B10
MAP
mtmB1
mtmC1
pylS1
pylB1
pylC1
pylD1
MAP
mtmC2
mtmB2
mtaC1
mtaB1
46.7 Kb
pylT
Contig 4
mta/ mtbA2
mta/mtbA1
mtaB3
MAP
mcrB
mcrD
mcrG
mcrA
mcrC
mtsB1
mtsA1
mtsA2
mtsB2
MAP
mtaC2
mtaB2
mtaC3
1.3 Kb
3 Kb
6 Kb
412 Kb
1.8 Kb
52.4 Kb
185 Kb
Contig 17
Contig 13
Contig 12
mtbC
mtbB
MAP
mtmB4
mtmC4
mttB
mttC
mttP
mtbP
pylS2
mtmB3
mtmC3
pylC3
pylB2
pylC2
pylD2
12.6 Kb
1 Kb
1.1 Kb
15.2 Kb
0.3 Kb
pylT
Pseudo pylT ?
Contig 23
Additional Figure S6
Physical mapping of genes involved in methanogenesis on methyl compounds + H2.
The distance (in Kb) between two genes is indicated when one or several genes not displayed on the map are situated between these two genes. The accession numbers of the genes presented on the map (except Pyl, MAP and the transporter genes) are given in Additional Table S10. MAP, Methyltransferase Activation Protein.
