# Supplementary material for: Comparative genomics highlights the unique biology of Methanomassiliicoccales, a Thermoplasmatales-related seventh order of methanogenic archaea that encodes pyrrolysine
Source: BMC Genomics. 2014 Aug 13;15:679. doi: 10.1186/1471-2164-15-679 (PMC4153887; doi:10.1186/1471-2164-15-679)
Supplement: Supplementary file 3 — Additional file 3: Additional Data in a zipped MS Word file. Details on lipids, amino acids and purine synthesis, as well as molecular nitrogen fixation deduced from the genomes of the three members of the Methanomassiliicoccales. (ZIP 26 KB) [file 12864_2014_6390_MOESM3_ESM.zip › 2014_BMCGenomics_Additional_Data.docx]

**Additional Data:**

**Lipid synthesis**

One of the important traits distinguishing the domain Archaea from the two other domains of life relies on the structural characteristics of their lipids [1]. Indeed, archaeal lipids contain hydrocarbon chains formed by methyl-branched isoprenoids (instead of straight-chain fatty acids), these chains are bound to the glycerol moiety by an ether linkage (instead of ester linkages) positioned in sn-2 and sn-3 of the glycerol moiety (instead of sn-1 and sn2), and in several archaeal orders, lipids consist of two C_40_ isoprenoid chains linking two glycerol moieties and form a monolayer membrane [1]. The building blocks of the hydrocarbon chains, the isopentenyl diphosphate (IPP) and its isomer dimethylallyl diphosphate (DMAPP), arise from the mevalonate (MVA) pathway [2-4].

Genes for the first 3 steps of the pathway, forming MVA with acetyl-CoA, are present in the 3 genomes, encoding the acetyl-CoA acetyltransferase (AGI85303.1, AGN26847.1, WP_019176549.1), the hydroxymethylglutaryl-CoA synthase (AGI85302.1, AGN26848.1, WP_019176548.1) and the 3-hydroxy-3-methylglutaryl-CoA reductase (AGI85530.1, AGN26207.1, WP_019177038.1). The subsequent steps of the MVA pathway are variable among archaea and all the enzymes are not yet identified and characterized. Two homologues of genes encoding mevalonate kinase (MVK) were identified in the three genomes, one is related to the gene occurring in most of archaea but not the Thermoplasmatales (AGI85105.1, AGN26642.1, WP_019177328.1) and the other is related to the recently described MVK specifically occurring in Thermoplasmatales among archaea [5] (AGI84828.1, AGN26686.1, WP_019178602.1). A mevalonate diphosphate decarboxylase (MDC, EC 4.1.1.33) was detected in *M. luminyensis* (WP_019177897.1), showing high sequence similarity with Halobacteriales MDC. MDC is detected only in Halobacteriales and Thermoplasmatales among archaea, and is supposed to originate via LGT from bacteria [6]. No CDS coding for this protein was found in the two other genomes. The phosphomevalonate kinase (PMK), absent in Euryarchaeota [4, 6] is also absent in the three genomes. A modified mevalonate pathway in *Methanocaldococcus jannaschii* was proposed by Grochowski *et al*. [7], where PMK and diphosphate mevalonate decarboxylase are replaced by a phosphomevalonate decarboxylase (PMDC) and an isopentenyl phosphate kinase (IPPK)*.* The same pathway may operate in the three species, as the genes encoding IPPK (AGI86174.1, AGN25923.1, WP_019176398.1) and the putative PMDC are present (AGI84914.1, AGN25566.1, WP_019177897.1).

An isopentenyl diphosphate isomerase (IDI2) is present for conversion of IPP into its isomerDMAPP (AGI86175.1, AGN25922.1, WP_019176397.1). These two isomers can be used as the building blocks for the synthesis of the hydrocarbon chain of lipids and respiratory quinones. The 3 genomes contain two different isoprenyl diphosphate synthases to condense DMAPP with several IPP to form polyprenyl diphosphate chains. One have the signature for short prenyl chain (<C_20_) synthesis (a bulky amino acid, phenylalanine, as the fifth amino acid residue before the first aspartate-rich motif) and is likely involved in the synthesis of the hydrocarbon chain of lipids [8] (AGI86176.1, AGN25921.1, WP_019176396.1). The other have the signature for long prenyl chain (>C_20_) synthesis (a small amino acid, alanine, as the fifth amino acid residue before the first aspartate-rich motif) and could be involved in the synthesis of the isoprenoids chain of membrane soluble electron transporters (AGI84964.1, AGN25614.1, WP_019176170.1). Supporting this hypothesis, long chain isoprenyl diphosphate synthase are only found in archaea that possess membrane soluble electron transporters [8] and the gene of this enzyme is adjacent to those encoding the respiratory complex I in the 3 Methanomassiliicoccales genomes. Other possible enzymes for the synthesis of the backbone of a membrane soluble electron carrier were detected in all three genomes, as were those for the transfer of the apolar head group on the backbone, but surprisingly not those coding for the synthesis of the apolar head group: therefore, the presence of a membrane soluble electron carrier remains putative, as does its real chemical nature.

Polyprenyl chains for lipid synthesis are binding with the glycerol moiety is accomplished by a geranylgeranylglyceryl phosphate (GGGP) synthase (AGI85008.1, AGN25972.1, WP_019176351.1) and a digeranylgeranylglyceryl phosphate (DGGGP) synthase for which two homologues are present in the *Methanomassiliicoccus* representatives (AGI85009.1, AGN25971.1, AGN26591.1, WP_019176352.1, WP_019178514.1). The DGGGP synthase of "*Ca.* M. alvus" is predicted to contain a pyrrolysine (see section on *amber* codon usage and putative Pyl-containing proteins).

**Amino acid auxotrophy**

Aromatic Amino Acid (AAA) synthesis originates from the shikimate pathway. It necessitates 3-dehydroquinate that is further converted into chorismate in a 5-step process. The required genes are clustered in an operon in the three genomes: the first two steps are catalyzed by a 3-dehydroquinate dehydratase (EC 4.2.1.10) and a shikimate dehydrogenase (EC 1.1.1.25). These two enzymes appear fused in a unique CDS sharing high similarity to 3-dehydroquinate dehydratase in its N-terminal coding part, while the part homologous to shikimate dehydrogenase is located in its C-terminal coding region (AGI85254.1, AGN26760.1, WP_019176508.1). Chorismate is further converted into tryptophan in a multi-step pathway while phenylalanine and tyrosine need first the synthesis of prephenate by a chorismate mutase (EC 5.4.99.5). The chorismate mutase enzyme differs among the three genomes: it is likely a bi-functional enzyme in *“Ca.* M. intestinalis” and *M. lumyniensis* (respectively 355 amino acids (AGN26764.1) and 394 amino acids (WP_019176504.1)) supporting also a prephenate dehydrogenase activity. In contrast, these enzymatic activities are separated in *“Ca.* M. alvus”, with the chorismate mutase being 97 amino acids long (AGI85237.1) and the prephenate dehydrogenase being 185 amino acids long (AGI85257.1). Several aminotransferases were detected, such as the aspartate aminotransferase (2 copies per genome) and the serine-pyruvate aminotransferase (EC. 2.6.2.52) SerC (AGI84920.1, AGN25589.1, WP_019178131.1), which may reversibly synthesize or use alanine. The gene *gltB* coding for the large subunit of glutamate synthase (EC 1.4.1.13) is present in the three genomes while the small subunit was not found, as in some other methanogen genomes [9]. In archaea, the large subunit is encoded by three juxtaposed CDSs (*gltB1-gltB2-gltB3*) in *“Ca.* M. intestinalis” (encoding AGN25622.1 to AGN25624.1) and *M. luminyensis* (encoding WP_019176157.1 to WP_019176159.1), and a second putative *gltB2* is also found at another different locus (encoding AGN26113.1, WP_019176854.1). By contrast, the glutamate synthase is encoded by a unique and long CDS in *“Ca.* M. alvus” genome (leading to AGI86324.1, a 1,463 amino acids long protein), as observed in bacteria, whereas no other *gltB* is detected*.* It has high sequence similarity with bacterial enzymes from the *Bacteroides* and *Parabacteroides* genera and likely reflects a LGT from these gut-associated bacteria, which may have been accompanied by a loss of the native archaeal genes. In conclusion, based on the genomic data, the three members of the Methanomassiliicoccales appear auxotrophic for amino acids. Some transporters likely support the entry of a few amino acids from the environment into the cell. These include at least uptake systems for histidine and proline, a methionine porter, symporters of Na^+^ with alanine and glutamate/aspartate, and a glutamine permease (Additional Table S8).

**Purine synthesis**

Purine synthesis shows variations among archaea, particularly concerning enzymatic activities converting aminoimidazole ribonucleotide (AIR) to carboxyaminoimidazole ribonucleotide (CAIR) (see [10] for a complete description of this pathway in archaea). In eukaryotes, this conversion is achieved directly by addition of CO_2_ by the (ATP-independent) class II PurE enzyme (AIR carboxylase, EC 4.1.1.21), whereas this is achieved by two enzymes in bacteria, first a N5-carboxyaminoimidazole ribonucleotide synthetase (NCAIR synthetase, PurK, EC 6.3.4.18) in an ATP-dependent reaction and then a NCAIR mutase (class I PurE) [37]. As in bacteria Thermoplasmatales, some Thermococcales, Halobacteriales, Thaumarchaeota and most Crenarchaeota possess a PurK and a class I PurE [10]. In contrast, in Archaeoglobales, the reaction is carried out by a class II PurE enzyme that likely results from an LGT from Eukarya [10]. In Methanobacteriales, the PurE homologue is longer, consisting of two fused PurE-like domains (PurE-PurE') which likely perform the activity of PurE class I and PurK [11]. Other methanogens have a class I PurE but no PurK. The three Methanomassiliicoccales genomes lack *purK* and present two different *purE*-like genes (AGI84793.1, AGI85002.1, AGN25661.1, AGN26431.1, WP_019178351.1, WP_019177087.1) which could not be assigned to class I or class II. The absence of PurK distinguishes the Methanomassiliicoccales from the Thermoplasmatales and suggests a process which could be closed to most of other methanogens for this step of the purine synthesis pathway. Absence of PurK is generally observed in archaeal species which thrive in CO_2_ rich environments. The production of CAIR without PurK and under high CO_2_ concentration has been demonstrated with an *E. coli* *purK* deletion and PurE overexpression [12]. The nature of the PurE enzymes is still unclear in the 3 Methanomassiliicoccales representatives, but a similar process relying on high CO_2_ concentrations might occur in the case that they only present a class I PurE activity.

**Molecular nitrogen fixation**

Fixation of molecular nitrogen relies on nitrogenase, a two component metalloenzyme composed of a dinitrogenase reductase and a dinitrogenase [13, 14]. The most common and well-studied nitrogenase is molybdenum-dependent (encoded by *nifH*, *nifD* and *nifK*). Two alternatives nitrogenases, a vanadium-dependent (encoded by *vnfH*, *vnfD*, *vnfG* and *vnfK*) and an iron-only dependent (encoded by *anfH*, *anfD*, *anfG* and *anfK*) were also described and only occur in microorganisms encoding the molybdenum-dependent nitrogenase [15]. *Methanomassiliicoccus luminyensis* genome presents the larger number of genes required for diazotrophy with two dedicated gene clusters, *nifHI_1_I_2_DKEN* (encoding WP_019178608.1 to WP_019178615.1) and *anfHI_1_I_2_KEN* (encoding WP_019177663.1 to WP_019177667.1). The putative gene of the AnfD subunit, situated between *anfH* and *anfG*, is interrupted by a stop codon and not deposited into Genbank. This gene interruption questions the effective activity of an iron-only dependent nitrogenase in *M. luminyensis*. Two copies of *nifB* are also present in *M. luminyensis* (encoding WP_019178691.1 and WP_019177392.1), one being associated to 2 genes putatively involved in the biosynthesis of a vanadium-dependent nitrogenase (*vnfEN*-like, encoding WP_019178693.1 and WP_019178693.1). In contrast, “*Ca.* M. alvus” and “*Ca.* M. intestinalis” do not contain the *anf* and *nif* gene cluster of *M. luminyensis* but only a *nifB* (encoding AGN25999.1, AGI85084.1). The three genomes contain genes coding for distant homologues of the core proteins required for diazotrophy such as 2 copies of a gene consisting in fused *nifH* and *nifE*-like genes encoding an uncharacterized protein also occurring in some representative of the Firmicutes, Deltaproteobacteria and few other methanogens. No homologue of nifK was found in “*Ca.* M. alvus” and “*Ca.* M. intestinalis”. Among the large and variable number of genes requested for N_2_ fixation, a core of 6 genes (*nifHDKENB*) is conserved among all known diazotrophs and was proposed by Dos Santos *et al.* [15] as a criterion for computational prediction of this metabolism. On this basis, *M. luminyensis* is predicted to be diazotroph with a putative flexibility upon the dependency on Mo, while “*Ca.* M. alvus” and “*Ca.* M. intestinalis” probably lack the capacity to fix N_2_. So far, none of the methanogen species strongly associated to digestive tracts (e.g. some *Methanobrevibacter* spp*.* or *Methanomicrobium* spp. [16, 17]) were described or predicted to be diazotrophs, contrasting with other species related to soils (e.g. *Methanocellales* spp*.* [18] or sediments (e.g. *Methanococcus spp.*([15] and references within). One explanation for this partitioning could relate on the high ammonium concentration in digestive tracts [19] that reduces the advantage of the diazotrophy and could even inhibit nitrogenase [20, 21]. Accordingly, the predicted N_2_ fixation capacity of *M. luminyensis* could reflect an adaptation to soil and suggests a facultative association to digestive tracts.

1. Koga Y, Morii H: **Biosynthesis of ether-type polar lipids in archaea and evolutionary considerations**. *Microbiol Mol Biol Rev* 2007, **71**(1):97-120.

2. De Rosa M, Gambacorta A, Gliozzi A: **Structure, biosynthesis, and physicochemical properties of archaebacterial lipids**. *Microbiol Rev* 1986, **50**(1):70.

3. Lange BM, Rujan T, Martin W, Croteau R: **Isoprenoid biosynthesis: the evolution of two ancient and distinct pathways across genomes**. *Proc Natl Acad Sci U S A* 2000, **97**(24):13172-13177.

4. Lombard J, López-García P, Moreira D: **Phylogenomic Investigation of Phospholipid Synthesis in Archaea**. *Archaea* 2012, **2012**.

5. Azami Y, Hattori A, Nishimura H, Kawaide H, Yoshimura T, Hemmi H: **(R)-mevalonate 3-phosphate is an intermediate of the mevalonate pathway in Thermoplasma acidophilum**. *J Biol Chem* 2014:jbc. M114. 562686.

6. Lombard J, Moreira D: **Origins and early evolution of the mevalonate pathway of isoprenoid biosynthesis in the three domains of life**. *Mol Biol Evol* 2011, **28**(1):87-99.

7. Grochowski LL, Xu H, White RH: **Methanocaldococcus jannaschii uses a modified mevalonate pathway for biosynthesis of isopentenyl diphosphate**. *J Bacteriol* 2006, **188**(9):3192-3198.

8. Villanueva L, Damsté JSS, Schouten S: **A re-evaluation of the archaeal membrane lipid biosynthetic pathway**. *Nat Rev Microbiol* 2014.

9. Hendrickson EL, Kaul R, Zhou Y, Bovee D, Chapman P, Chung J, Conway de Macario E, Dodsworth JA, Gillett W, Graham DE *et al*: **Complete genome sequence of the genetically tractable hydrogenotrophic methanogen Methanococcus maripaludis**. *J Bacteriol* 2004, **186**(20):6956-6969.

10. Brown AM, Hoopes SL, White RH, Sarisky CA: **Purine biosynthesis in archaea: variations on a theme**. *Biol Direct* 2011, **6**:63.

11. Hamilton PT, Reeve JN: **Sequence divergence of an archaebacterial gene cloned from a mesophilic and a thermophilic methanogen**. *J Mol Evol* 1985, **22**(4):351-360.

12. Patrick WM, Quandt EM, Swartzlander DB, Matsumura I: **Multicopy suppression underpins metabolic evolvability**. *Mol Biol Evol* 2007, **24**(12):2716-2722.

13. Howard JB, Rees DC: **Structural basis of biological nitrogen fixation**. *Chem Rev* 1996, **96**(7):2965.

14. Orme-Johnson W: **Molecular basis of biological nitrogen fixation**. *Annu Rev Biophys Biophys Chem* 1985, **14**(1):419-459.

15. Dos Santos PC, Fang Z, Mason SW, Setubal JC, Dixon R: **Distribution of nitrogen fixation and nitrogenase-like sequences amongst microbial genomes**. *BMC genomics* 2012, **13**(1):162.

16. Gaci N, Borrel G, Tottey W, O'Toole PW, Brugere JF: **Archaea from the human gut: the new beginning of an old story**. *World J Gastroenterol* in press.

17. Janssen PH, Kirs M: **Structure of the archaeal community of the rumen**. *Appl Environ Microb* 2008, **74**(12):3619-3625.

18. Conrad R, Erkel C, Liesack W: **Rice Cluster I methanogens, an important group of *Archaea* producing greenhouse gas in soil**. *Curr Opin Biotechnol* 2006, **17**(3):262-267.

19. Macfarlane GT, Gibson GR, Cummings JH: **Comparison of fermentation reactions in different regions of the human colon**. *J Appl Bacteriol* 1992, **72**(1):57-64.

20. Cejudo F, De la Torre A, Paneque A: **Short-term ammonium inhibition of nitrogen fixation in Azotobacter**. *Biochem Biophys Res Commun* 1984, **123**(2):431-437.

21. Leigh GJ: **Nitrogen fixation at the millennium**: Elsevier; 2002.
